# Targeted Protein Degradation in Cancer: PROTACs, New Targets, and Clinical Mechanisms

**DOI:** 10.3390/biom16020325

**Published:** 2026-02-19

**Authors:** Bushra Faryal, Zain Ul Abideen, Muhammad Irfan, Haseeb Ahmed, Fazliddin Jalilov, Lola Abduraximova, Ghulam Abbas Ashraf

**Affiliations:** 1 Department of Precision Medicine, University of Campania “Luigi Vanvitelli”, 80138 Naples, Italy; 2 Department of Biosciences, COMSATS University Islamabad, Islamabad 45550, Pakistan; 3 The Department of Pharmaceutical and Chemistry, Alfraganus University, Tashkent 100190, Uzbekistan; 4 Kimyo International University in Tashkent, Shota Rustaveli str. 156, Tashkent 100121, Uzbekistan; 5 Low Dimensional Materials Research Center, Khazar University, Baku AZ1096, Azerbaijan

**Keywords:** PROTACs, cancer, targeted protein degradation, E3 ubiquitin ligase, next-generation cancer therapeutics

## Abstract

The onset of proteolysis targeting chimeras (PROTACs) has reshaped the entire context of targeted cancer therapy by offering a novel approach for the selective degradation of disease-causing proteins, overcoming the limitations of traditional occupancy-driven inhibition. This heterobifunctional technology recruits endogenous E3 ubiquitin ligases to mark proteins of interest (POI) for proteosomal degradation via the ubiquitin-proteasome system (UPS). Unlike conventional inhibitors, PROTACs function catalytically and can target previously “*undruggable proteins*”, such as transcription factors, scaffold proteins, and non-enzymatic regulators, offering potential to overcome acquired resistance and achieve potent efficacy at sub-stoichiometric doses. The review explores the latest innovations in PROTAC design, including E3 ligase selection, linker chemistry, and ligand optimization, while highlighting promising preclinical and clinical candidates against oncogenic drivers, anti-apoptotic factors (BCL-xL), and nuclear hormone receptors. Furthermore, we critically examine key translational challenges, such as pharmacokinetics, off-target effects, and resistance mechanisms, and discuss viable solutions, including dual E3 ligase engagement, novel modalities like AUTACs/ATTECs, LYTACs, and AI-driven design. As the field rapidly evolves from foundational to clinical application, PROTACs are redefining therapeutic possibilities, offering a robust, flexible, and scalable framework for the future of precision oncology.

## 1. Introduction

Despite recent breakthroughs in clinical practice, cancer remains a leading cause of morbidity and mortality worldwide [1]. Factors such as a poor lifestyle, demographic aging, and discrepancies in the healthcare system contribute to its growing burden, with ~20 million new cancer cases and 9.7 million deaths reported globally in 2020, and an increase to 35 million new cases projected by 2050. The most common cancers, including breast, lung, and colorectal cancers, pose significant challenges in both developed and developing nations due to limitations in treatment access, diagnostic, and preventive strategies [2]. Beyond mortality, cancer imposes significant economic and social burdens, including lost productivity, high treatment costs, and reduced quality of life for long-term survivors [3]. Although targeted therapies and immunotherapies have improved outcomes for certain cancers, numerous malignancies remain refractory to treatments due to intrinsic and acquired drug resistance, tumor heterogeneity, and a limited range of druggable targets [4].

A principal limitation of conventional small molecule inhibitors is their reliance on occupancy-driven mechanisms, which require high systemic exposure and continued target binding, often leading to off-target toxicity and selective pressure that drives resistance [5]. Moreover, a vast portion of the proteome, including transcription factors, scaffold proteins, and non-catalytic effectors, has been considered *undruggable* due to the absence of deep enzymatic pockets or functional binding sites [6]. These unresolved challenges underscore the urgent need for innovative therapeutic modalities that can overcome the limitations of current anticancer treatments [7].

Proteolysis targeting chimeras (PROTACs) represent a revolutionary class of heterobifunctional molecules that exploit the cell’s natural ubiquitin-proteasome system (UPS) to achieve selective protein degradation. A typical PROTAC consists of two ligands: one that binds a protein of interest (POI), and another that recruits an E3 ubiquitin ligase, connected by a chemical linker. By facilitating proximity-induced ubiquitination, PROTACs catalyze the degradation of the target protein, offering several advantages over traditional inhibitors, including sub-stoichiometric activity, ability to target non-enzymatic proteins, and potential resilience against resistance mutations.

For example, PROTACs such as *ARV-471 (targeting ER-alpha)* and *ARV-110 (targeting AR)* have shown promising clinical activity in hormone-resistant breast cancer and prostate cancers, where standard therapies fail [8].

Recent advances have expanded the PROTAC toolbox to include conditional activation strategies, tumor-specific delivery systems, and novel E3 ligase recruits. However, challenges remain in optimizing pharmacokinetics, minimizing on-target off-tissue toxicity, and achieving tissue-selective degradation.

In this review, we aim to provide a comprehensive analysis of the current landscape of PROTAC technology, focusing on its mechanistic basis, design innovations, and clinical progress. We further discuss persistent challenges in translation and highlight emerging solutions such as covalent PROTACs, dual targeting degraders, and integrative artificial intelligence approaches that promise to enhance the specificity, efficacy, and applicability of PROTACs. By synthesizing recent preclinical and clinical advances, this review offers a forward-looking perspective on the role of PROTACs in overcoming the limitations of conventional therapy and expanding the frontiers of precision oncology.

## 2. Overview of PROTAC Design

**Figure 1:**
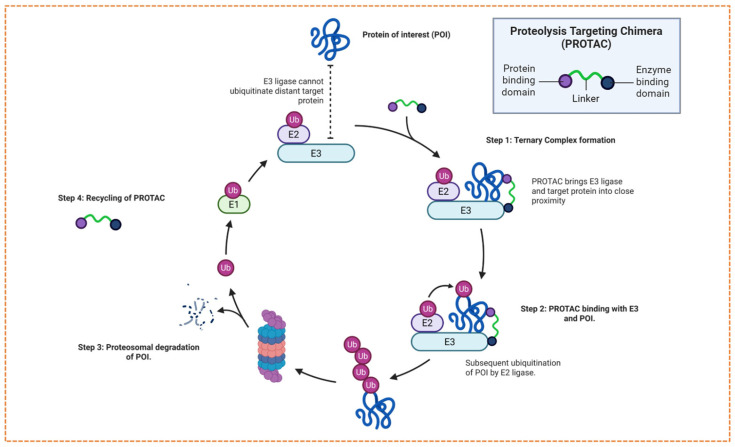
Overview of PROTAC design and mechanism of action, utilizing the cell proteasome system, and PROTAC-mediated protein degradation. PROTACs simultaneously bind a POI and an E3 ubiquitin ligase, facilitating the transfer of ubiquitin (UB) from the E2 enzyme to the POI. The polyubiquitinated POI is then recognized and degraded by the proteasome. The PROTAC is released and recycled to initiate further degradation cycles.

PROTACs consist of heterobifunctional molecules that contain three essential structural components: a ligand that binds to the POI, a ligand that attaches to an E3 ubiquitin ligase, and a chemical linker that connects these two parts, as shown in Figure 1. Target ligands are often derived from binding fragments or traditional small-molecule inhibitors, and their primary function is to bind specifically to the POI with high affinity and selectivity [9].

E3 ligases are the enzymes that assist *CRBN* and *VHL*-based ligands in the ubiquitin process by tagging or recruiting target proteins for degradation by proteasomes [10]. Among the large number of E3 ligases, cereblon (CRBN) and von Hippel-Lindau (VHL)have emerged as the most used in PROTAC development due to the availability of well characterized small molecule binders and their consistent ability to induce efficient target degradation across diverse proteins.

**Figure 2:**
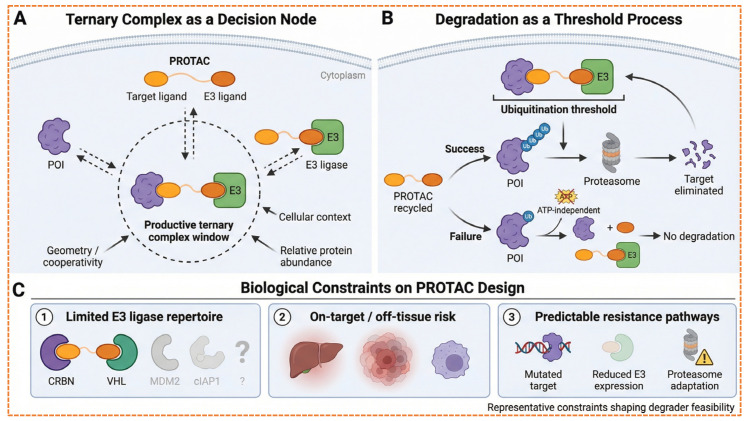
Conceptual overview of PROTAC mechanism and design. (**A**) PROTAC-mediated ternary complex formation, in which a bifunctional molecule simultaneously binds a protein of interest and an E3 ligase via a target-binding ligand and an E3 ligase connected by a linker. (**B**) Recruitment of the ubiquitination machinery results in polyubiquitination of the target protein, followed by proteasomal degradation. (**C**) Examples of E3 ligases commonly used in PROTAC design development including CRBN, VHL, MDM 2 and Ciap1.

Careful selection of the E3 ligase is crucial to the degradation mechanism because it impacts degradation efficiency, tissue distribution, and the potential for drug resistance. Additionally, the composition of linker molecules is a significant determinant of 3D protein structure and the structural plasticity required during the formation of the ternary complex involving all components, including POI, PROTAC, and E3 ligase. Any changes in linker properties, such as linker length, attachment chemistry, and rigidity, significantly affect PROTACs’ effectiveness, including degradation potency and pharmacokinetics [11]. Recent developments in PROTAC technology have led to the integration of a conditional active mechanism into linker design, providing greater control over selectivity and protein degradation Figure 2. These modifications in linker design allow PROTACs to respond to or be activated by specific intracellular conditions like pH, redox status, and light. Optimizing PROTACs is challenging due to their modular design, which involves complex requirements for solubility, molecular size, and cellular permeability [12].

### 2.1. Mechanism of Action

**Figure 3:**
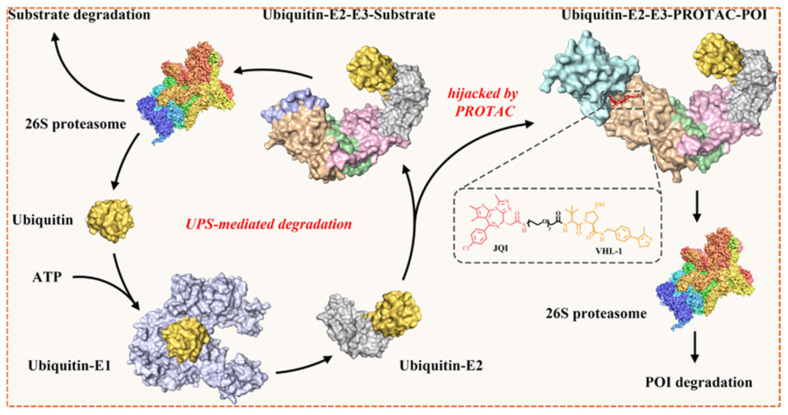
The mechanism of PROTACs based on the UPS. UPS consists of specific enzymes (E1, E2, and E3) modifying proteins with ubiquitin, and the proteasome degrading the ubiquitin-tagged proteins. PROTAC hijacked the UPS machinery and formed an E3-PROTAC-POI ternary complex. This induces the polyubiquitination and proteasome-mediated degradation of POIs. Reproduced with the permission of [14]. Copyright 2022 Springer Nature.

PROTACs technology utilizes the cell’s UPS to specifically degrade targeted proteins within the cells, as shown in Figure 3. They achieve this by binding the POI in closer proximity to an E3 ubiquitin ligase, forming an interim ternary complex [13]. Upon formation of the ternary complex, the E3 ligase promotes polyubiquitination of the target protein, thereby tagging it for subsequent degradation by the 26S proteosome [14]. After the ubiquitin-tagged target is degraded, the proteolysis chimera molecule is released with its catalytic activity intact and is now available to bind with another target and E3 ligase, continuing additional proteolytic cycles [15]. The main difference between PROTACs and traditional small inhibitors is that they do not constantly rely on the active site; instead, they employ an event-driven approach for targeted degradation. This mechanism enables PROTACs to reduce endogenous protein concentrations below stoichiometric amounts [16].

A detailed understanding of the PROTAC mechanism of action relies on a combination of complementary and Analytical approaches. Biophysical and structural techniques, including surface plasmon resonance, isothermal titration, calorimetry, and cryo-electron microscopy, are increasingly used to characterize ternary complex formation and stability. Cellular assays such as immunoblotting, reporter-based degradation assays, and live cell imaging enable quantitative assessment of target degradation kinetics and durability. In parallel, ubiquitination profiling and proteomics-based approaches provide insights into selectivity, off target effects and pathway level responses. Together, these methodologies are essential for linking greater design to biological outcome and for guiding rational optimization of PROTAC efficacy and safety.

### 2.2. Differences Between Small Traditional Inhibitors and PROTACs

**Figure 4:**
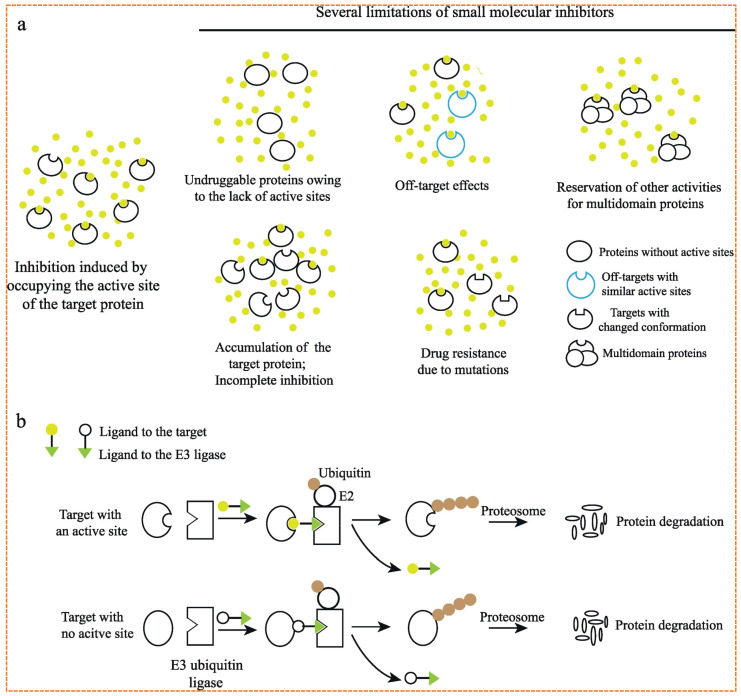
Overview of the mechanisms of small molecule inhibitors and PROTACs. (**a**) To inhibit the activities of target proteins, small-molecule inhibitors competitively bind to active sites on the target proteins. The limitations on developing and taking small-molecule drugs are shown in this figure; (**b**) Heterobifunctional PROTAC molecules harness the ubiquitin proteasome system to selectively degrade target proteins. Currently, the generation of PROTACs relies on available small molecular inhibitors to be used as target-binding ligands. Alternatively, PROTACs can bind to any binding site on the surface of the target proteins to induce their degradation. Reproduced with the permission of [20]. Copyright 2018 Elsevier.

PROTACs provide a powerful alternative to small-molecule inhibitors through an event-driven approach that results in the gradual and controlled breakdown of targeted proteins instead of merely blocking their activity temporarily [17]. Traditional inhibitors usually function by occupying the allosteric site (active site) of a protein, resulting in the blocking of its biological function, as shown in Figure 4. This inhibition often depends on high-affinity, long-lasting interactions with the target to maintain effectiveness. Conventional small-molecule inhibitors are less effective against mutated proteins with inactive active sites and non-enzymatic roles [18]. In contrast, PROTACs can trigger the irreversible degradation of the protein of interest (POI) via the UPS within cells. Their catalytic nature enables PROTACs to produce therapeutic effects at levels below stoichiometric amounts, decreasing the need for continuous target engagement [19].

The advantage of PROTACs is their ability to target proteins lacking enzymatic activity, such as scaffolding proteins, transcription factors, and regulatory subunit targets that are typically considered “undruggable” with standard inhibition techniques. Furthermore, because PROTACs eliminate proteins rather than just inhibiting their function, they may evade resistance mechanisms associated with point mutations that diminish the binding affinity of inhibitors [14]. Nevertheless, challenges related to large molecular weight, less oral bioavailability, and limited cellular permeability still need to be addressed for effective clinical implementation [21]. However, PROTACs present a powerful and versatile approach to managing proteins therapeutically across a broad spectrum of disease targets, especially in the field of oncology (Table 1).

**Table 1:** Major differences between PROTACs and traditional small-molecule inhibitors with distinct benefits and challenges associated with Targeted Protein Degradation.

Feature	PROTACs	Traditional Inhibitors	Ref.
**Mechanism of Action**	Induce degradation via the ubiquitin–proteasome system.	Block the enzymatic or functional activity.	[22]
**Target Engagement**	Event-driven (Transient)	Occupancy driven (Continuous)	
**Target Type**	Scaffolding proteins (enzymatic and non-enzymatic)	Mostly enzymatic	[23]
**Catalytic Behavior**	Degradation of multiple copies of the same target protein	Only 1:1 target inhibitor binding is required	[24]
**Reversibility**	Irreversible target degradation	Mostly reversible target degradation	[25]
**Selectivity**	Higher due to ternary complex formation	Depends on the Inhibitor binding specificity	[26]
**Resistant Potential**	Ability to overcome target resistance due to point mutation	Susceptible to resistance from a binding site mutation	[27]
**Drug-like Properties**	Complex and larger, with permeability issues	Smaller and drug-like compounds	[28]
**Therapeutic Duration**	Prolonged and steady effect due to protein removal	Limited effect due to binding duration	[5]
**Clinical Stage**	Under trials and Emerging	Clinically approved and widely used.	

### 2.3. Role of E3 Ligases in PROTAC Design

**Figure 5:**
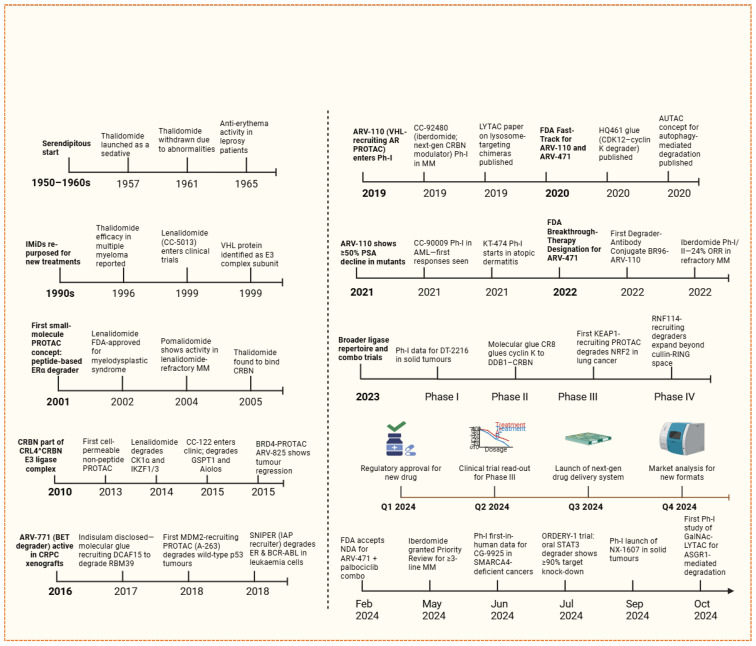
Updated Timeline (1950s–2024) of key milestones in the development of PROTAC technology over the years with expansion of novel E3 ligases and degrader modalities.

E3 ubiquitin ligases play a crucial role in designing PROTACs, as they facilitate the ubiquitination and gradual proteasomal degradation of target proteins. The effectiveness of PROTACs relies on the formation of a productive ternary complex that includes the target protein, the PROTAC compound, and the recruited E3 ligase. This process is influenced by the biochemical and cellular characteristics of the E3 ligase, such as its ligand-binding capacity, expression across various tissues, subcellular localization, and compatibility in protein–protein interactions (PPI) [29]. More than 600 E3 ligases are present in the human genome; however, only 2% are utilized in the development of PROTACs, including cereblon (*CRBN*), von Hippel-Lindau (*VHL*), *MDM2*, and inhibitors of apoptosis, due to their known small-molecule ligands and advantageous structural properties. In Figure 5, a timeline of successful E3 ligases for drug discovery and development is shown.

E3 ubiquitin ligases showed higher degradation efficiency, stable ternary complex formation, and applicability across a wide range of targeted classes of proteins. For instance, *CRBN* and *VHL* have been extensively utilized due to their potent ligands and comprehensive validation in preclinical studies [30]. The choice of E3 ligase depends on various aspects such as degradation efficiency, subcellular localization, and tissue specificity [31]. There are broad opportunities to explore new ligases, like *FBXO7*, *HUWE1*, and *UHRF1*, because of their specific expression in tumors and their ability to target proteins that were previously considered “undruggable,” such as *KRAS* or *RAC1* [32]. New datasets, like the *E3 Atlas*, combine multi-omics information, for instance, ligand ability, PPIs, structure, and expression, to identify ligases that can be prioritized for the next generation of PROTACs [33]. Furthermore, exploiting substrate-ligase interactions and known PPIs extends the part of the degradable proteome. For example, integrating ligases, such as *ITCH*, *SKP2*, or *SMURF2*, facilitates the degradation of cancer-related targets like *NRAS* and *EGFR* [34]. Simultaneously using an approach to design PROTAC with dual ligands that hold two ligases together, for example, *CRBN* and *VHL*, has demonstrated a tenfold increase in degradation efficiency and a hundredfold increase in cytotoxicity in vitro, proposing a method to overcome resistance or redundancy in ligase pathways [35]. In conclusion, the careful choice of E3 ligases is crucial for the efficacy, selectivity, and therapeutic promise of PROTACs [36]. Future advancements will rely on investigating less commonly used ligases, developing covalent ligase recruiters, and combining structural and expression information to design degraders with enhanced specificity and lesser off-target effects [37].

### 2.4. Recent Innovations in PROTAC Design

In recent years, the limitations of traditional PROTACs, such as suboptimal pharmacokinetics, off-target effects, and limited tissue selectivity, have encouraged the development of a diverse array of next-generation targeted protein degradation (TPD) strategies to enhance their clinical potential [38]. One of the main advancements is the development of *reversible covalent PROTACs* as a major innovation that combines the benefits of both reversible and irreversible binding, offering improved target occupancy time and catalytic efficiency while maintaining reversibility for enhanced safety [39]. Compared to covalent PROTACs, which irreversibly bind either the target protein or the E3 ligase, are showing promise for degrading traditionally “*undruggable*” targets, though their irreversible binding can limit catalytic turnover and pose challenges for clinical translation [40]. In comparison, *Molecular glue degraders*, are a unique approach represented by *CC-885* (*CRBN* binding molecules) and *lenalidomide*, have highlighted their relevance, particularly in hematologic malignancies, by stabilizing transient E3 ligase substrate interactions and a neo substrate using monovalent molecules, expanding the range of targetable proteins [41]. *Trivalent PROTACs* (*TriTACs*), which introduced a third binding domain to stabilize ternary complexes and enhance selectivity, degradation potency, especially for multiple functional domain proteins such as the *BET* family [42]. *PHOTACs* (photoactivable PROTACs) have been developed to achieve spatiotemporal control over degradation using a specific wavelength, using light-sensitive linkers to trigger protein degradation with precise spatial and temporal resolution, offering precision for treating localized or dynamically regulated diseases such as *glioblastoma* and *melanoma* [43]. Another innovation is the development of *Conjugates of Antibody-PROTAC* (*AbTACs*) linking monoclonal antibodies with PROTACs for receptor-mediated delivery to specific targeted cells, thereby reducing systemic toxicity with enhanced selectivity and expanding the therapeutic options [44].

**Figure 6:**
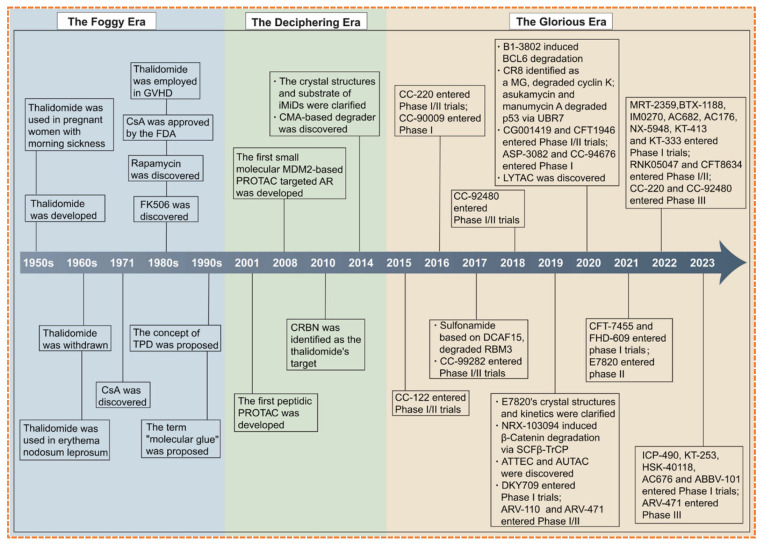
Timeline of the development of targeted protein degradation technology. The timeline spans three key eras: the Foggy Era, marked by Thalidomide’s rise and withdrawal, CsA discovery, and early unclear TPD use; the Deciphering Era, defined by the formal TPD concept and crystal structure–based mechanistic insights; and the Glorious Era, characterized by rapid clinical progress, novel pathways like *LYTAC*, and transformative potential across multiple diseases. Reproduced with the permission of [46]. Copyright 2022 Nature.

In parallel, the field has also begun to target RNA with novel degraders such as *RIBOTACs*, which use *RNase L* to degrade specific RNA transcripts, thereby extending the degradation role beyond proteins. Additionally, in-cell click chemistry approaches now enable the intracellular assembly of PROTACs to enhance bioavailability and minimize off-target effects through localization. Their activity is directed at specific cellular compartments. Collectively, these innovations are driving a role shift in TPD as given in Figure 6, opening new avenues for overcoming resistance, improving selectivity, and expanding the druggable proteome across a wide spectrum of diseases [45].

## 3. Clinical Landscape and Trials of PROTACs in Cancer

The rapid advancement of PROTAC technology has accelerated translational research, with multiple candidates initially validated in an academic setting now progressing toward the pharmaceutical development pipeline [47]. This marks a significant transition from theoretical innovation to practical application, particularly in oncology. As we look toward 2025, several PROTACs targeting well-defined oncogenic drivers, notably nuclear hormone receptors *AR, ER*, and anti-apoptotic *BCL-xL*, have entered Phase I and II clinical trials, led by companies like Arvinas, Dialectic Therapeutics, and Kymera Therapeutics [48]. Leading this clinical forefront are *ARV-110* and *ARV-471*, which target the androgen and estrogen receptors, respectively. Both have shown clinical promise, an acceptable safety profile, and initial effectiveness in phase II trials. Other compounds like *DT2216* (targeting *BCL-xL*) and *NX-2127* (targeting *BTK*) are broadening the therapeutic range of PROTACs to include hematologic cancers and solid tumors [49]. However, alongside these advancements, challenges such as pharmacokinetics, resistance mechanisms, and biomarker identification remain crucial factors for improving cancer treatment.

A careful analysis of these properties of these clinical and preclinical candidates provides valuable insights into their effectiveness in cancer therapy, as summarized in (Table 2 and Table 3).

**Table 2:** Comparative Clinical Profile of PROTACs vs. Standard of Care Inhibitors.

PROTAC (Trial ID)	SOC Agent	Key Efficacy Endpoint (PROTAC vs. SOC)	Key Safety Advantage (PROTAC)	Key Safety Concern (PROTAC)	Resistance Context and Biomarkers	Ref.
**ARV-110 (NCT03888612)**	Enzalutamide	PSA50 in AR T878X/H875Y+: ~46% (ARV-110) vs. <10% (enzalutamide)	Manageable GI toxicity (Nausea ~42%) vs. higher fatigue/seizure risk with enzalutamide	Nausea, fatigue	Overcomes common AR ligand-binding domain mutations (T878A, H875Y)	[50,51]
**ARV-471 (NCT04072952)**	Fulvestrant	CBR in heavily pre-treated ER+: ~40% (ARV-471) vs. ~20% (fulvestrant in similar population); >90% ER degradation	Oral bioavailability vs. IM injection; potentially lower incidence of musculoskeletal pain	Fatigue, nausea	Active against ESR1 mutants; biomarker: ERα levels by imaging	[52,53]
**DT2216 (NCT04886622)**	Navitoclax	Efficacy in T-cell lymphomas without dose-limiting thrombocytopenia (DT2216) vs. severe thrombocytopenia (navitoclax)	VHL-based tissue specificity spares platelets	To be fully characterized in trials	Exploits low VHL expression in platelets for a wider therapeutic window	[54,55]
**NX-2127 (NCT04830137)**	Ibrutinib/Acalabrutinib	BTK degradation in CLL patients with BTK C481 mutations; early signs of efficacy in BTKi-resistant disease	Dual action (BTK + IKZF1/3 degradation)	Neutropenia, fatigue (CRBN-related)	Overcomes covalent (C481) and non-covalent (PLCγ2, BTK kinase domain) resistance mechanisms	[56,57]

**Table 3:** Selected PROTACs in Preclinical and Clinical Development for Cancer Treatment.

PROTAC	Target Protein	E3 Ligase	Cancer Type/Indication	Developmental Phase	Trial Identified Number	Company	Ref.
***ARV-110***	Androgen Receptor (AR)	VHL	Metastatic Castration-Resistant Prostate Cancer (mCRPC)	I/II(ongoing)	NCT03888612	Arvinas	[58]
***ARV-471***	Estrogen Receptor α (ERα)	VHL	ER+/HER2− Breast Cancer	II (with Pfizer) III (VERITAC-2)	NCT04072952 NCT05654623	Arvinas	
***DT2216***	BCL-xL	VHL	Hematological malignancies, solid tumors	I	NCT04886622	Dialectic Therapeutics	[59]
***KT-474***	IRAK4	CRBN	Immuno-inflammatory diseases (RA, HS)	I	NCT04772885	Kymera Therapeutics	[60]
***NX-2127***	BTK, IKZF1/3	CRBN	B-cell malignancies (CLL, NHL)	I	NCT04830137	Nurix Therapeutics	
***CFT7455***	IKZF1/3	CRBN	Multiple Myeloma, Non-Hodgkin Lymphoma	I/II	NCT04756726	C4 Therapeutics	
***ARV-393***	BCL6	Unknown	Diffuse Large B-cell Lymphoma (DLBCL)	Preclinical	N/A	Kymera Therapeutics	[61]

### 3.1. ARV-110: An Androgen Receptor (AR) Degrader for Prostate Cancer

**Figure 7:**
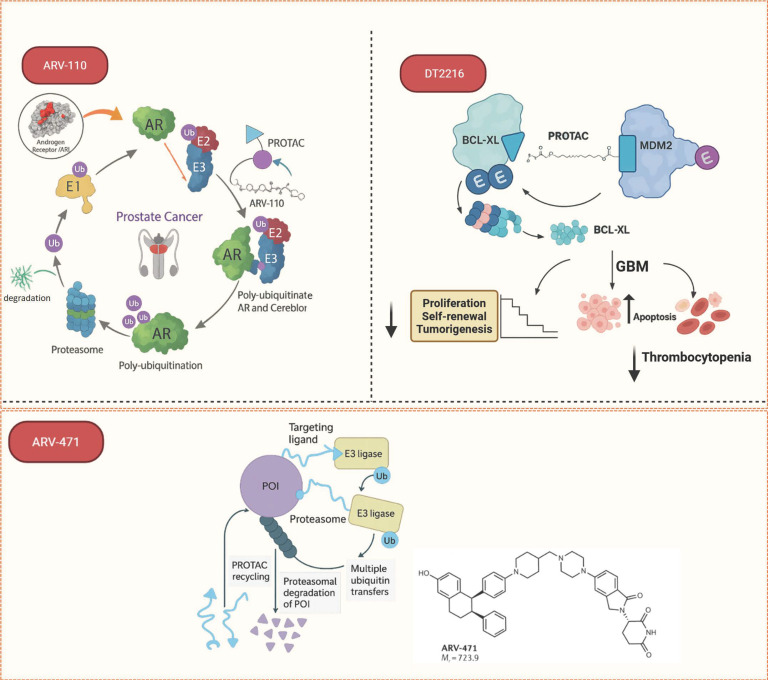
The mechanistic overview of selected clinical-stage PROTACs. It shows four representative PROTAC molecules currently in clinical development: ARV-110 (targeting AR in prostate cancer), ARV-471 (targeting ERα in breast cancer), and DT2216 (targeting BCL-xL in hematologic and solid tumors).

Bavdeglutamide (*ARV-110*) is an orally bioavailable PROTAC degrader designed to target the androgen receptor (AR) for the treatment of metastatic castration resistant prostate tumors (*mCRPC*). It consists of an AR-targeting ligand connected via a short, rigid linker to a ligand for the *CRBN* E3 ubiquitin ligase, facilitating the formation of a productive ternary complex that leads to AR ubiquitination and proteasomal degradation (Figure 7) [62].

Clinical evaluation in the Phase I/II trial (NCT03888612) has demonstrated its promising profile. In heavily pre-treated *mCRPC* patients, *ARV-110* showed a favorable safety profile and dose-proportional pharmacokinetics, supporting a recommended dose of 420 mg administered once daily [62]. Its efficacy is particularly notable in patients with specific AR ligand-binding domain mutations. *ARV-110* induced a PSA50 response (a ≥50% decline in prostate-specific antigen) in 46% of patients bearing AR T878X/H875Y mutations, with tumor reduction observed in 6 out of 7 evaluable patients and two confirmed partial responses [63]. This underscores its potential to overcome resistance to standard antiandrogens by degrading clinically relevant AR mutants.

The most common treatment-related adverse events were nausea (42%), fatigue (27%), and decreased appetite (19%). The safety profile was deemed manageable, with no grade or higher toxicities reported at the recommended dose. Based on these encouraging phase I/II results, *ARV-110* is being further evaluated in combination with standard therapies like abiraterone and enzalutamide [64].

### 3.2. ARV-471: An Estrogen Receptor Alpha (ERα) for Breast Cancer)

*ARV-471* (Vepdegestrant) is an orally bioavailable PROTAC degrader specifically designed to target estrogen receptor alpha (ERα) for the treatment of ER+/HER− breast cancer. Developed in collaboration with Pfizer, it aims to overcome resistance to endocrine therapies. Its mechanism of action involves a ligand targeting ERα connected to a *VHL* E3 ligase recruiter, facilitating the formation of a stable ternary complex that leads to the proteasomal degradation of ERα (Figure 7). *ARV-471* has shown superior degradation efficiency and anti-proliferative activity compared to *fulvestrant*, the current selective estrogen receptor degrader (SERD) [65]. In initial clinical trials, it showed impressive selectivity, achieving over a 90% reduction in tumor ER levels, as measured by imaging and circulating biomarkers. Data from Phase I/II trials indicated a clinical benefit rate of approximately 40%, with observed partial responses in heavily pre-treated patients [52].

A key advantage of ARV-471 is its favorable safety profile, which reports minimal off-target toxicities, compared to existing SERDs like *fulvestrant* [66]. Its oral bioavailability and once-daily dosing regimen further underscore its promise as a next-generation endocrine therapy. Based on this strong monotherapy activity, ARV-471 is currently undergoing advanced phase II/III trials and is under investigation in combination with CDK4/6 inhibitors [67].

### 3.3. DT2216: A BCL-xL Degrader for Hematologic and Solid Tumors

*DT2216* is a PROTAC molecule engineered to target and degrade *BCL-xL*, a key anti-apoptotic protein overexpressed in various hematologic malignancies and solid tumors. It achieves this by utilizing the *VHL* E3 ligase ligand, a strategic choice that confers a critical therapeutic advantage. The primary challenge in targeting *BCL-xL* is on-target, off-tumor toxicity; inhibition causes dose-limited thrombocytopenia because platelets require *BCL-xL* for survival (Figure 7).

*DT2216* overcomes this major limitation of traditional inhibitors like navitoclax by exhibiting low endogenous expression of VHL in platelets. This creates a therapeutic window: *DT2216* effectively degrades BCL-xL in tumor cells (which express VHL) while sparing platelets, thereby minimizing thrombocytopenic toxicity and achieving tumor-selective apoptosis.

This principle of tissue-restricted degradation has been validated in early clinical assessments. In phase I trials (NCT04886622), *DT2216* demonstrated indications of apoptosis induction in patients without causing significant platelet toxicity [68]. Initial clinical activity has been observed in T-cell lymphomas and myeloid leukemias. The ongoing trial continues to focus on dose optimization and identification of predictive biomarkers to guide future developments [69].

### 3.4. KT-474: An IRAK4 Degrader for Immuno-Inflammatory Cancers

**Figure 8:**
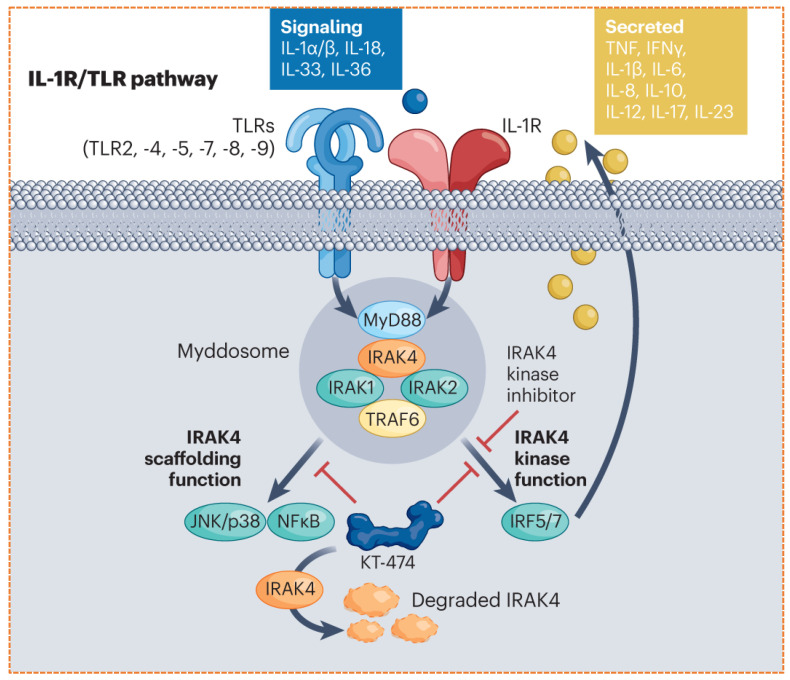
Role of IRK4 in IL-1R/TLR signaling and its modulation by targeted degradation. Overview of IL-1 receptor (IL-1R) and Toll-like receptor (TLR) signaling pathways, highlighting the leading role of IRAK4 within the Myddosome complex. Upon receptor activation, MyD88 recruits IRAK4, IRAK1/2, and TRAF6, which drives downstream activation of NF- kB and MAPK (JNK/p38) signaling and promotes the production of pro-inflammatory cytokines. Inhibition or targeted degradation of IRAK4 interferes with both its kinase activity and scaffolding function. Thereby attenuating downstream signaling and cytokine release. IRAK4 degradation enables broader suppression of inflammatory signaling compared with kinase inhibition alone. Reprinted with the permission of [71]. Copyright 2023 Springer Nature.

*KT474* is a PROTAC degrader that specifically targets interleukin-1 receptor-associated kinase 4 (IRAK-4), a key node in innate immune and NF Kappa B signaling pathways [70]. Unlike other therapies, it functions through the recruitment of the *CRBN* E3 ligase. Although initially created for treating autoimmune inflammatory diseases (e.g., hydrocodone suppurativa, atopic dermatitis), its mechanism of action shows significant potential in cancers characterized by overactive NF-κB signaling. In preclinical studies, *KT-474* has demonstrated the ability to selectively degrade IRAK4 and subsequently suppress pro-inflammatory cytokines, reinforcing the rationale for its further exploration in oncology and immunotherapy (Figure 8) [71]. Its progression into clinical trials for inflammatory indications provides valuable safety and pharmacokinetic data that can inform its potential application in cancers.

### 3.5. NX-2127: A Dual BTK and IKZF1/3 Degrader for B-Cell Malignancies

**Figure 9:**
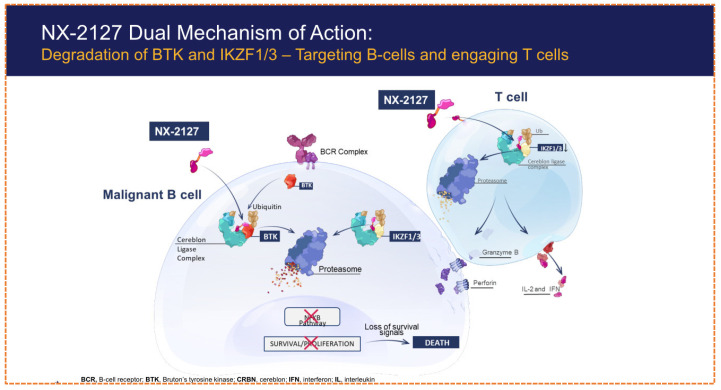
NX-2127 mechanism of action in malignant B-cell malignancies. NX-2127 recruits the cereblon E3 ligase complex to induce proteasomal degradation of both BTK and IKZF1/3, disrupting BCR signaling and transcriptional programs needed for cell survival and proliferation. In T cells, NX-2127-mediated IKZF1/3 degradation promotes enhanced immune activity through granzyme B and IL-2 secretion, further contributing to malignant cell death. Reproduced with the permission of [72].

NX-2127 is an oral, first-in-class, dual-function, small-molecule degrader that combines BTK degradation with the immunomodulatory activity of a degrader for the transcription factor Ikaros (IKZF1/3) (Figure 9). An innovative class of dual degraders designed to target both Bruton’s tyrosine kinase BK and the transcription factors Ikaros (IKZF1) and Aiolos (IKZF3) through the recruitment of the *CRBN* E3 ligase. It is specifically designed for patients with relapsed/refractory chronic lymphocytic leukemia (CLL) and other B-cell malignancies, particularly those resistant to both covalent and non-covalent *BTK* inhibitors.

**Figure 10:**
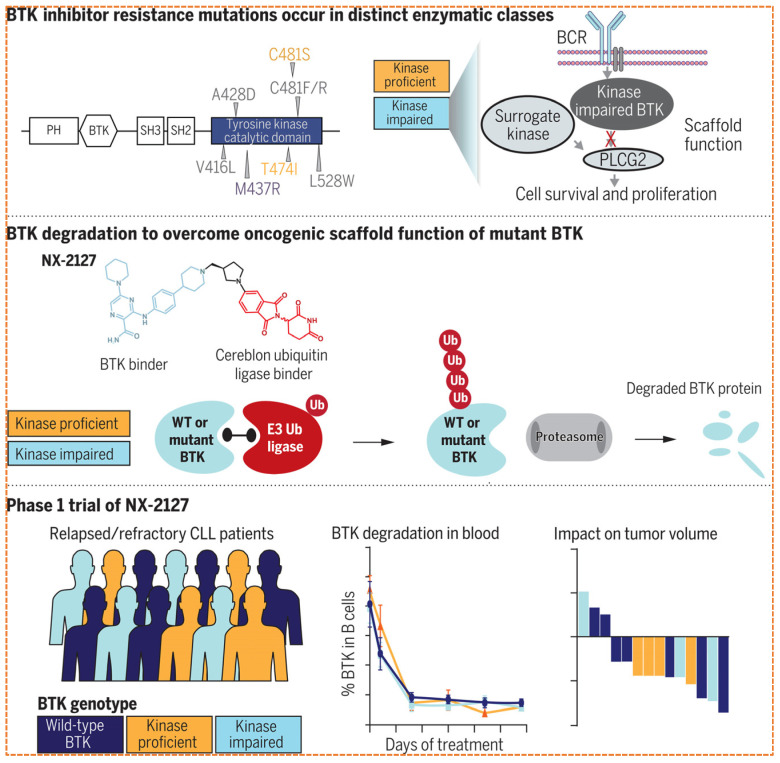
NX-2127 induces degradation of BTK and IKZF1/3, overcoming resistance from kinase-impaired BTK mutations. Reprinted with the permission of [74]. Copyright 2024 American Association for the Advancement of Science.

In early-phase clinical trials, pharmacokinetic and pharmacodynamic evaluations confirmed the persistent and simultaneous degradation of both *BTK* and IKZ F1/3 [73]. Clinical activity has been observed even in this treatment-resistant population, with outcomes including disease stabilization in lymph node size Figure 10 and Table 4. The side effect profile has been manageable and in line with the known class of facts for CRBN-modulating agents, such as neutropenia and fatigue. Future research directions include investigating NX21207 in combination with BCL-2 inhibitors like Venetoclax [72].

## 4. Challenges and Limitations in Clinical Translation of PROTACs

**Figure 11:**
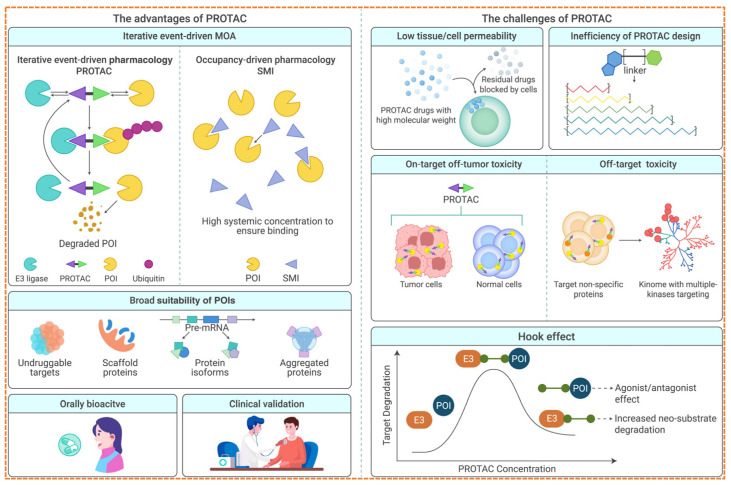
Key limitations and challenges associated with PROTAC-based therapeutics. Summary of principal factors that can constrain PROTAC efficacy and translational potential. Excessive PROTAC concentrations can favor the formation of non-productive binary complexes with either the target protein or the E3 ligase, thereby impairing ternary complex formation and reducing degradation efficiency (Hook effect). Additional limitations include inefficient ternary complex stability, limited cellular permeability, heterogeneity in E3 ligase expression, and Pharmacokinetic constraints, and the risk of off-target protein degradation. Reproduced with the permission of [81].

Although PROTACs hold significant promise for TPD, various challenges confine their smooth transition into clinical use. These challenges include pharmacological, biochemical, and developmental areas, requiring a multidisciplinary approach to overcome them [80] as given in Figure 11.

One well-recognized limitation of PROTAC-based strategies is the so-called hook effect, in which excessive PROTAC concentrations reduce degradation efficiency rather than increasing it. At high concentrations, PROTAC molecules preferentially form binary complexes with either the target protein or the E3 ligase, which competes with productive ternary complex formation required for ubiquitination [82]. As a result, optimal degradation is often observed within a narrow concentration window, highlighting the importance of careful dose optimization during PROTAC development.

### 4.1. Pharmacokinetics and Bioavailability Restrictions

One of the most significant translation barriers is the suboptimal pharmacokinetic (PK) inherent to PROTAC molecules. Their high molecular weight (often greater than 900 Da), substantial polar surface area, and heterobifunctional design often lead to poor membrane permeability, limited oral bioavailability, and rapid systemic clearance [83]. As summarized in Table 5, clinical-stage PROTACs such as ARV-110 (MW ~1000 Da, clogP ~6.59) and DT-2216 (MW ~1100 Da, clogP ~7.1) exemplify these challenges, possessing physicochemical properties that clearly violate Lipinski’s rule of five and complicate formulation. Clinical data reveal these properties directly impact PK; for example, an *ARV-110* exhibits a short half-life (~3.6 h), necessitating high daily doses (420 milligrams QD) to maintain exposure, while other degraders show low oral absorption [NCT03888612]. To address these limitations, strategies such as prodrug designs, nanoparticle-based delivery platforms, and alternative administration routes (e.g., subcutaneous, intratumoral injections) are under active investigation to improve bioavailability and therapeutic index [84].

### 4.2. E3 Ligase Availability and Expression in Specific Tissues

The effectiveness of PROTACs depends on the expression and activity of the recruited E3 ligase. Presently, the research area is focused on *CRBN* and *VHL*, both of which have limited expression patterns in various tissues. For example, the low levels of *VHL* in specific hematopoietic cells, such as platelets, may reduce efficacy or reduce toxicity, depending on the context. Furthermore, alterations or decreased expression of *CRBN* have been associated with acquired resistance, especially in patients with multiple myeloma who have been treated with *CRBN*-recruiting degraders.

### 4.3. Off-Target Effects and Proteome-Wide Degradation

PROTACs are designed for specific targets, but off-target effects can arise from non-specific interactions between E3 ligases and off-targeted proteins, potentially resulting in cytotoxic effects, changes in immune responses, or interference with off-targeted signaling pathways [49]. Since PROTACs promote protein degradation rather than merely inhibiting their function, the removal of multifunctional proteins can create complex and nonspecific cellular responses. The level of selectivity depends on the stability of the ternary complex and the interaction networks involving the recruited E3 ligase [85]. To enhance the safety and efficacy of PROTACs in clinical applications, it is crucial to increase the diversity of E3 ligases, optimize linker designs, and conduct comprehensive evaluations of off-target effects [51].

### 4.4. Emerging Resistance Mechanisms and Strategies to Overcome Them

Resistance to PROTAC therapies, akin to traditional targeted treatments, can arise through various mechanisms [86]. These include mutations in the target protein that sterically hinder PROTAC binding or ternary complex formation to downregulation or functional modifications of the recruited E3 ligase (e.g., altered CRBN functionality after prolonged CRBN-based PROTAC administration), impairment of the ubiquitin proteasome system components, and activation of compensatory adaptive cellular pathways [87]. Although PROTACs offer a significant advantage by degrading entire proteins, including mutant forms, resistance remains a critical translation challenge [19].

This is exemplified in well-established resistance pathways for targets like *BTK* and the androgen receptor (AR), where resistance often arises from point mutations, overexpression, and structural changes that diminish drug binding capacity. To address these barriers, innovative PROTAC-specific strategies are being developed, including [88]:

Dual targeting degraders: molecules designed to simultaneously engage multiple functional sites or critical nodes in a protein pathway to preempt escape mechanisms [89].

Allosteric targeting: the ability to bind to allosteric sites, enabling degradation even after mutations occur in the canonical binding pockets [46].

Rescue of weak binders: converting partial antagonists or weak binding molecules into potent degraders, resurrecting efficacy against resistant cancer cells.

The clinical potential of these strategies is beginning to be realized. For instance, the *MDM2-based* PROTAC *KT-253* has demonstrated a significant increase in potency compared to traditional *MDM2* inhibitors, achieving durable tumor regression in preclinical models [90]. Furthermore, the advancement of candidates like CC-94676 and ARV-471 into late-stage clinical trials provides robust validation for the ability of TPD to overcome resistance [91].

Beyond molecular design, next-generation delivery approaches are being explored to circumvent resistance. This includes the development of stimulus-responsive nano PROTACs activated by exogenous (e.g., light ultrasound) or endogenous (e.g., enzymes, pH) triggers to achieve spatiotemporal control over degradation, thereby minimizing off-target toxicity and potentially overcoming bioavailability-related resistance. The expansion of PROTAC modalities into non-oncological indications, such as viral infections, further highlights their versatile potential to overcome the limitations of conventional therapeutics [92].

### 4.5. Chemical and Manufacturing Complexity

The clinical translation of PROTAC has unequivocally established that their chemical synthesis and manufacturing represent a significant advancement in complexity over conventional small molecules, forming a major non-biological barrier to their commercial viability. This complexity, inherent to their heterobifunctional architecture, manifests across four critical domains that impact yield, stability, and scalability, from milligram to Good Manufacturing Practices (GMP) kilogram scale [93].

(1) Linker instability and reliability: The linker is a critical vulnerability. Common spacers like PEG chains, alkyl esters, and carbonates are prone to degradation via hydrolysis, β-elimination, or oxidative scission, especially during late-stage coupling reactions under basic or acidic conditions [94]. This leads to batch instability out of out-of-specification failures, and short shelf lives. Recent data (2022 to 2024) show a strategic shift towards more robust amide/urea linkages and the implementation of rigorous stress testing protocols for linker fragments under process conditions. Furthermore, the widespread adoption of prodrug strategies (e.g., phosphate or peptide masking) serves a dual purpose, improving solubility and overcoming linker liability by design, with activation occurring only intracellularly [95].
(2) Chirality, Isomerism, and Atropoisomeric: the convergence of multiple stereocenters from complex E3 and target ligands with new chiral centers introduced during linker attachment creates complex isomeric mixtures. Different diastereomers and atropisomers can exhibit orders of magnitude differences in degradation potency, pharmacokinetics, and toxicity [96]. This complicates purification and analytical release, necessitating resource-intensive chiral chromatography for separation. control strategies now emphasize chiral pool synthesis and asymmetric catalysis early in the route scouting phase to minimize this burden. Clinical stage examples have proven that failing to control chirality can lead to variable efficacy and is a major GMP challenge [97].
(3) Low Yield Steps and Impurity Profiles: specific chemical transformations are consistent yield bottlenecks. amide couplings with sterically hindered linkers, click chemistry (CUAAC), and final deprotection steps often proceed in low yields, less than 30%, generating complex impurity profiles including over-acylated PEGs, oligomers, and ligand degradants [98]. Modern process chemistry addresses this through the design of experiments (DoE) to optimize coupling reagents and the adoption of continuous flow reactors. flow chemistry, particularly for exothermic or hazardous reactions like CuAAC, provides superior heat/mass transfer, reduces impurity formation, and enhances safety and reproducibility at scale [99].
(4) Scale-Up and GMP Compliance Hurdles: the high molecular weight, amphiphilic nature, and structural flexibility of PROTAC cause severe scale-up issues; poor solubility in GMP-acceptable solvents (necessitating solvents swaps from DMF/DMAc), difficult filtration, and resistance to crystallization [100]. These often result in amorphous solids with batch-to-batch variability, campaign restarts, and clinical material shortages. Mitigation strategies include generating stable salt/cocrystal forms and employing spray-dried dispersions to ensure consistent bioavailability [101]. For GMP release, establishing robust orthogonal analytical methods (e.g., qNMR, LC-MS/MS, chiral HPLC) is essential to characterize complex impurities and ensure identity, purity, and stability [102].

While innovations in automated synthesis, flow chemistry, and biocatalysis are improving yields and control, the synthesis and manufacturing of PROTACs remain a leading cause of supply delays, attrition, and clinical programs. The field is increasingly adopting a “developability-first” mindset where synthetic tractability and scalability are prioritized alongside potency early in the discovery process [103]. Success relies on deep collaboration between medicinal and process chemists to design synthetically accessible motifs and on partnerships with specialized CDMOs to navigate the challenging route from discovery to have a consistent, stable, and scalable GMP drug product [104] Table 6.

### 4.6. Immunogenicity and Systemic Effects of PROTACs

PROTACs experience difficulties related to immunogenicity and systemic effects because of their bifunctional structure and dependence on the UPS. The following is a summary of principal factors and potential risks derived from recent studies:

#### Immunogenicity Risks

- Recognition of Foreign Molecules: The synthetic composition of PROTACs, which includes non-human E3 ligase ligands such as *VHL or CRBN*, may trigger immune responses, particularly in non-oncology contexts that require long-term dosing. While clinical data on immunogenicity is currently unavailable, recombinant antibody-based PROTACs (*AbTACs*), designed with humanized components, aim to reduce this risk [57].
- Interference with E3 Ligase Substrates: *CRBN*-based PROTACs risk degrading native substrates, such as *IKZF1/3*, which are essential for immune cell function. For instance, the neutropenia observed in *CFT7455* trials may be related to the spontaneous degradation of these proteins [46].
- Presentation of Neoantigens: The degradation induced by PROTACs can enhance the presentation of tumor neoantigens via *MHC-I*, potentially improving anti-tumor immune responses. Conversely, excessive exposure to antigens may lead to autoimmune-like reactions (Table 7) [111].

## 5. Emerging Innovations in PROTAC Therapeutics

**Figure 12:**
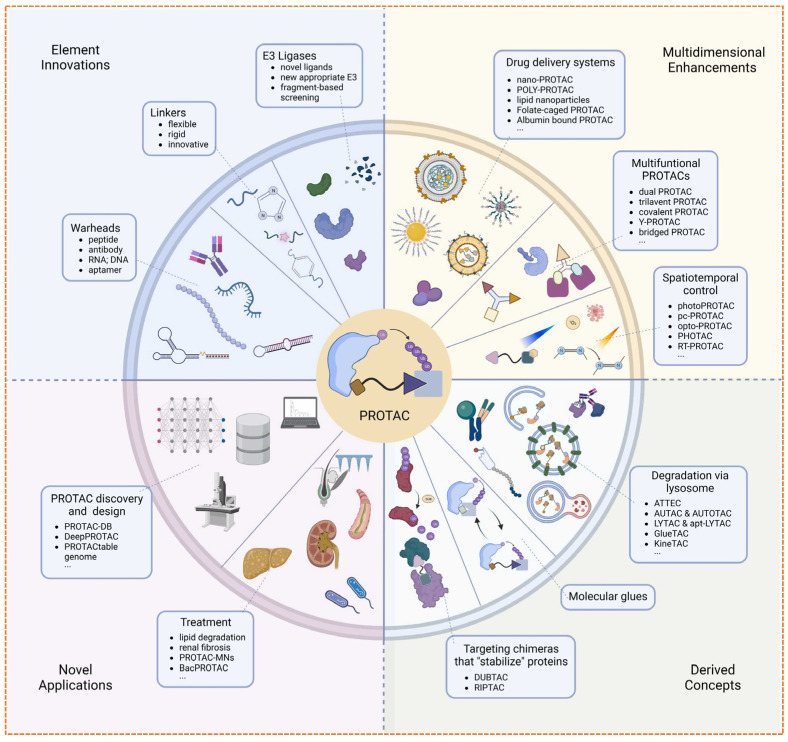
Summary of PROTAC-related innovative technologies with the perspectives of element innovations, combinations with new techniques, technological extension, and new applications. Reprinted with the permission of [118]. Copyright 2024 Elsevier.

The development of PROTACs has moved from a theoretical idea to a clinically proven approach. With initial trials exhibiting target interaction and clinical effectiveness, the future aims to tackle existing challenges while broadening the scope of therapies [117]. Numerous innovations are transforming the forthcoming generation of degradation-based treatments as shown in Figure 12.

### 5.1. Expanding the E3 Ligase Toolbox

**Figure 13:**
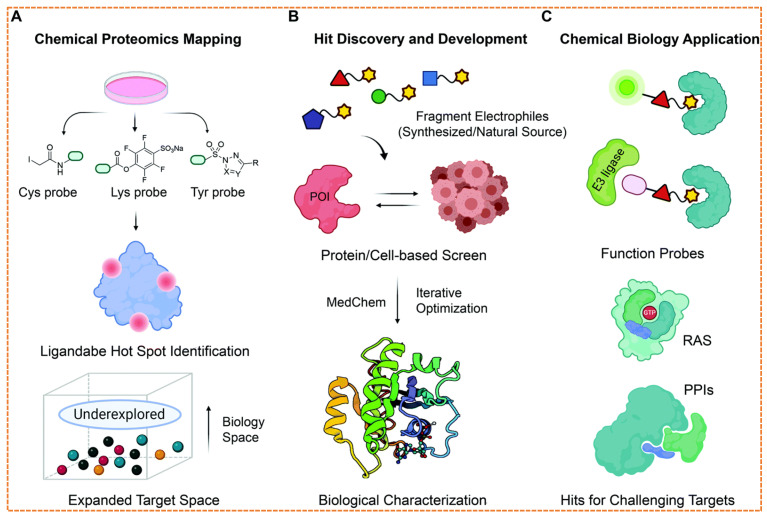
The roadmap for fragment-based covalent ligand discovery. (**A**) State-of-the-art chemoproteomic strategies help to systematically unveil potential ligandable sites in disease-associated targets. Cells are treated with reactive fragments (biased towards thiols (Cys), amines (lysine) and phenols (tyrosine)), and then chemoproteomics allows identification of proteins and reaction sites. (**B**) Fragment-based covalent ligand screening identified covalent fragment hits, which can be evolved into more potent, selective, biocompatible and drug-like ligands by iterative elaboration and optimization. (**C**) Fragment-based ligand discovery pipeline holds great promise as an initial ligand-discovery approach that can be elaborated to make bivalent molecules that can recruit other enzymes, including E3s, for PROTACs. Reproduced with the permission of [121]. Copyright 2021 Royal Society of Chemistry.

The clinical efficacy of PROTAC is intrinsically linked to the recruited E3 ubiquitin ligase. However, the field remains heavily reliant on a narrow subset predominantly cereblon (CRBN) and von Hippel-Lindau (VHL), which together account for over 90% of reported degraders [119]. This dependence creates critical vulnerabilities: a limited number of degradable targets, potential for on-target off-tissue toxicity, and a single mechanistic route to resistance, such as CRBN downregulate or mutation in multiple myeloma [120]. The human genome encodes over 600 E3 ligases, yet fewer than 2% have been leveraged effectively in targeted protein degradation (TPD), representing a vast untapped resource for overcoming these limitations [33]. The expansion of this toolbox is now being driven by systematic chemoproteomic and computational strategies. Techniques like isotopic tandem orthogonal proteolysis activity-based protein profiling (isoTOP-ABPP) enable the screening of covalent fragment libraries against reactive cysteine residues, identifying lead compounds for ligases such as DCF 16 and RNF114 (Figure 13) [107,113].

Concurrently, integrative computational platforms like the E3Atlas and the “PROTACtable genome” project are systematically prioritizing E3s based on multi-omics criteria: ligand ability, tissue-specific expression, essentiality, and protein–protein interaction networks (Table 8) [111,112]. This data-driven approach has nominated over 70 high-confidence E3 ligases as viable candidates for degradation.

However, the path from ligand discovery to a functionalized, clinical-grade E3 recruiter is full of challenges. A primary bottleneck is the transition from a covalent fragment hit to a highly selective, potent, and drug-like ligand that does not impair the native activity of the E3 complex [125]. Furthermore, many novel ligases lack well-characterized tissue-specific expression or natural substrate profiles, making it difficult to predict on-target-off tissue toxicity [126]. For instance, while RNF114 overexpression in squamous cell carcinomas offers a theoretical therapeutic window, its role in immune regulation poses a potential risk for immune-related adverse events. Similarly, recruiting KEAP1 in lung cancer is mechanistically appealing, but requires exquisite ligand specificity to avoid unintended stabilization of its natural substrate, NRF2, and activation of antioxidant pathways [127].

Tissue specificity remains a critical design goal. Ligases like SKP2 (overexpressed in triple-negative breast cancer) and KHL20 (defined expression in solid tumors) offer opportunities for cancer-selective degradation. The concept of dual ligase PROTAC, which simultaneously engages 2 different E3s (e.g., CRBN and VHL), presents another innovative strategy to enhance degradation efficacy and overcome resistance by redundantly activating the ubiquitin proteasome system [128]. In summary, expanding the E3 ligase toolbox is not merely a chemical exercise but a strategic imperative for the next generation of TPD. Success will depend on leveraging chemoproteomics for ligand discovery, computational tools for intelligent E3 prioritization, and a clear-eyed understanding of the therapeutic index and translational challenges inherent to each new ligase recruiter pair [129].

### 5.2. Emerging Degrader Modalities (Beyond PROTACs)

While conventional bifunctional PROTACs dominate the TPD landscape, several innovative degrader modalities are emerging to overcome their limitations and expand the scope of targetable proteins. These include molecular glues, trivalent PROTACs, and systems hijacking lysosomal degradation pathways [130]. Each platform offers distinct advantages but also faces unique challenges, as summarized in Table 9.

### 5.3. Lysosomal Targeting Chimeras (LYTACs)

**Figure 14:**
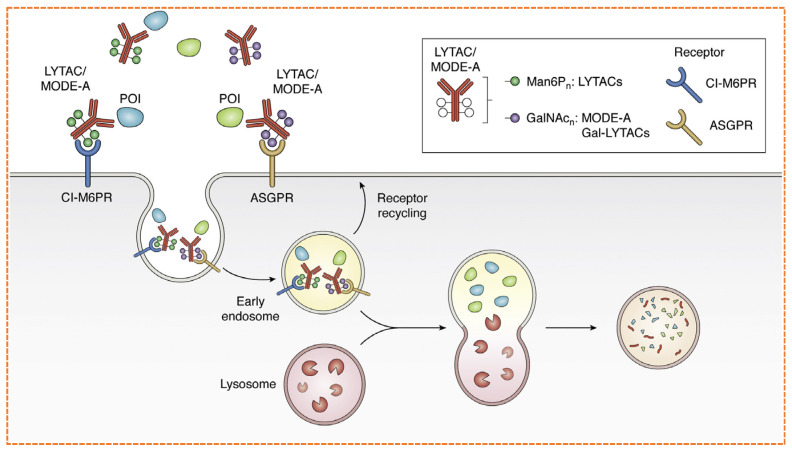
Co-opting the endosomal-lysosome pathway with LYTACs and MoDE-As. LYTACs and MoDE-As tether extracellular or membrane-bound POIs to a recycling receptor, which facilitates internalization and subsequent lysosomal degradation. LYTACs utilize the cation-independent mannose-6-phosphate receptor CI-M6PR, a recycling membrane protein that binds POIs labeled with an MP6n tag. GalNAc-LYTACs and MoDE-As utilize a GalNAC tag to recruit the asialoglycoprotein receptor (ASGPR), a liver-specific lysosomal targeting receptor, for tissue-specific degradation. Reprinted with the permission of Copyright 2021 American Society for Biochemistry and Molecular Biology.

Approximately 40% of the human proteome comprises extracellular proteins, including cytokines, growth factors, and proteins that play a crucial role in disease progression. Because the Ubiquitin proteosome system is present intracellular, for molecular glues and PROTACs, it is inaccessible to target proteins that are present in the extracellular environment. To target extracellular proteins, the Bertozzi lab developed Lysosomal-Targeting Chimeras (LYTACs) (Figure 14).

The development of LYTACs has opened a new prospect for TPD, especially for membrane-associated proteins that were previously inaccessible to PROTACs. A concept-based study by the Bertozzi group has shown that cation-independent mannose-6-phosphate receptor (CI-M6PR), a recycling receptor presents in the trans Golgi network, assimilate for endolytic delivery of extracellular proteins to the lysosome.CI-M6PR naturally interacts with ligands that carry a mannose-6-phosphate (M6P) tag and transports them to lysosomes, where the acidic environment promotes the release and breakdown of the cargo. By attaching synthetic mannose-6-phosphonate (M6Pn) groups to antibodies at serine or lysine sites, the researchers developed LYTACs that can trigger the lysosomal breakdown of target proteins after internalization via receptor-mediated endocytosis [135]. This was confirmed using biotinylated fluorescent streptavidin as a model substrate, and further mechanistic evidence was provided through a CRISPR screen, which pinpointed the exocyst complex and CI-M6PR as crucial elements [136]. Notably, the team applied this approach to disease-relevant targets, such as apolipoprotein E4 (ApoE4) linked to Alzheimer’s disease and the epidermal growth factor receptor (EGFR), achieving more than 70% degradation of EGFR within 24 h using cetuximab-M6Pn LYTACs. The degradation effect was maintained for at least 72 h, showcasing its long-lasting impact. Additionally, they achieved degradation of PD-L1 by up to 70% using antibody-directed LYTACs, indicating potential uses in immuno-oncology. Expanding on this concept, the Spiegel group developed Molecular Degraders of Extracellular Proteins through the Asialoglycoprotein Receptor (MoDE-As), which utilizes ASGPR, a receptor that is highly expressed in the liver and characterized by its rapid recycling properties and affinity for galactose and N-acetylgalactosamine (GalNAc) [137]. Their small-molecule design is modular and includes a trivalent GalNAc ligand connected to dinitrophenol (DNP) through a PEG-based spacer, allowing for the creation of a ternary complex with DNP-specific antibodies. This complex is efficiently trafficked to lysosomes and undergoes degradation, as indicated by its co-localization with LAMP2-positive compartments. The effectiveness of the MoDE-A system was further validated by the in vivo degradation of the cytokine MIF, highlighting its therapeutic potential [138].

Follow-up research conducted by the Tang and Bertozzi teams broadened the strategies focused on ASGPR, demonstrating that GalNAc-conjugated LYTACs achieve more effective internalization and degradation compared to CI-M6PR-based methods, with notable reductions in surface EGFR levels at considerably lower concentrations. Both cetuximab-GalNAc and cetuximab-M6Pn LYTACs produced similar maximal degradation levels, underscoring the platform’s versatility. Additionally, incorporating integrin-binding peptides with tri-GalNAc ligands led to significant integrin depletion and inhibited cancer cell growth. Importantly, the attachment of tri-GalNAc to small molecules (such as biotin) or antibodies effectively initiated lysosomal degradation, with improved internalization efficiency noted for smaller protein substrates [139]. In summary, these investigations assert that LYTACs and similar ligand-based systems are valuable tools to broaden the degradable proteome beyond intracellular targets. Like the critical role played by E3 ligase ligands in PROTAC technology, the future development of LYTACs will hinge on identifying more recycling receptors and ligands with specific tissue expression, allowing for wider and more selective therapeutic uses [140].

However, the translational path for LYTACs is full of challenges. Antibody-based constructs pose a significant risk of immunogenicity, particularly with chronic dosing regimens required for cancer therapy. Their large size limits effective tissue penetration into solid tumors [141]. Furthermore, the dependence on specific recycling receptors restricts their application: the GalNAc-ASGPR system is primarily effective for liver-specific targeting, while CI-M6PR is ubiquitously expressed, raising concerns for on-target off-tissues toxicity. The synthetic complexity of producing homogeneous, well-characterized antibody-oligosaccharide conjugates at scale also presents a major manufacturing hurdle that must be overcome for clinical translation [142].

### 5.4. Macroautophagy Degradation Targeting Chimeras (MADTACS): AUTACs/ATTECs

While PROTACs and other platforms that depend on the UPS have transformed the field of intracellular protein degradation, their dependence on this system limits their use to soluble, cytosolic proteins [143]. On the other hand, autophagy [144], a degradation pathway mediated by lysosomes, provides an appealing and adaptable option capable of removing aggregated proteins, dysfunctional organelles, and even non-protein biomolecules [145]. In recent years, several innovative chimeras targeting autophagy have been developed, each utilizing various components of the autophagic machinery to broaden the range of proteins that can be degraded [146].

The initial strategy, known as Autophagy-Targeting Chimeras (AUTACs), utilizes a distinctive degradation tag that draws inspiration from S-guanylation, a posttranslational modification found during xenophagy to mark targets for targeted autophagic degradation [147]. Created by the Arimoto group, AUTACs feature this S-guanylation mimic (p-fluorobenzylguanine, FBnG) attached to ligands that specifically interact with the proteins of interest. This chimeric structure promotes the recruitment of ubiquitin and autophagy receptors, such as p62/SQSTM1, which facilitates the degradation in lysosomes. AUTACs have proven effective at degrading both soluble proteins like MetAP2 and FKBP12, as well as damaged mitochondria in conditions relevant to diseases, such as fibroblasts from individuals with Down syndrome [148]. Notably, tagging mediated by FBnG has also been observed to trigger mitophagy through the degradation of outer mitochondrial membrane proteins, including TSPO. Nevertheless, despite their versatility, AUTACs typically necessitate high micromolar concentrations to attain sufficient degradation, and the specific molecular mechanism behind FBnG-induced autophagy is still not completely understood [147].

In addition to AUTACs, Autophagy Tethering Compounds (ATTECs) employ a “molecular glue” strategy to link the protein or organelle of interest to LC3, a key element of autophagosome membranes. Unlike AUTACs, ATTECs do not rely on ubiquitination; instead, they directly attach to both LC3 and the target substrate. The Lu group’s research significantly uncovered small-molecule ATTECs that can selectively degrade mutant huntingtin (mHTT) while leaving wild-type HTT unharmed, by taking advantage of the polyglutamine-rich regions unique to mHTT. Compounds such as 10O5, AN1, and AN2 exhibited effective degradation in cellular and animal models of Huntington’s disease, without causing any noticeable toxicity [149]. Furthermore, ATTECs that target lipid droplets (LDs), organelles that store lipids and are associated with hepatic steatosis and metabolic disorders, have succeeded in nearly entirely clearing LDs in vitro and murine models, further confirming the versatility of the platform. Nonetheless, the development of ATTECs is currently constrained by the lack of appropriate LC3 and POI-binding components, necessitating further structural enhancements to broaden their target range [150].

A novel strategy has been developed utilizing AUTOTACs (Autophagy-Tethering Chimeras), which are bifunctional compounds that can simultaneously attach to the target protein and the ZZ domain of the autophagy receptor *p62*. Once bound, *p62* undergoes oligomerization, leading to the sequestration and transport of the target protein into the autolysosome for degradation [151]. AUTOTACs have demonstrated efficacy in the degradation of aggregation-prone proteins linked to neurodegenerative disorders and oncogenic drivers in cancer research models [152]. These compounds operate at nanomolar concentrations and are capable of degrading monomeric, oligomeric, and aggregated forms of proteins. However, there are still unresolved questions concerning their potential catalytic properties, lysosomal recycling mechanisms, and selectivity profiles, which are critical factors for future applications in translational research [153].

**Figure 15:**
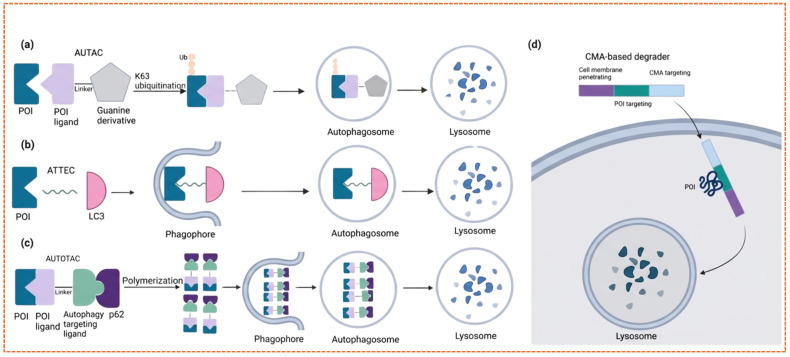
The principles behind degrader technologies that exploit autophagy pathways are outlined in the following sections. (**a**) AUTACs attach to the POI and link a degradation tag that simulates S-guanylation, a post-translational modification that activates K63 ubiquitination of the POI. This modification enables the autophagy receptor SQSTM1/p62 to recognize the POI and facilitate its recruitment into the selective autophagy pathway for degradation. (**b**) ATTECs bind simultaneously to the POI and LC3, effectively anchoring the POI to either the phagophores or autophagosomes, which leads to its autophagic degradation. (**c**) In AUTOTACs, the primary mechanism involves the p62-binding ligand activating the otherwise inactive p62 conformationally, transforming it into a version compatible with autophagy. When the ligand binds to the p62 portion, it reveals PB1 and LIR domains, thereby enhancing p62 self-polymerization in conjunction with targets and its interaction with LC3 on autophagic membranes. (**d**) The CMA-based degrader infiltrates the cell, then connects with the target protein using the POI binding sequence before being directed to the lysosome for degradation. Reproduced with permission of [112]. Copyright 2025 American Chemical Society.

In addition to systems based on macroautophagy, Chaperone-Mediated Autophagy (CMA) has also been utilized for specific degradation. CMA-targeted chimeras comprise three functional components: a cell-penetrating peptide, a ligand that binds the POI, and a CMA-targeting motif such as the *KFERQ* sequence. These compounds have demonstrated efficacy in degrading cytosolic proteins within neuronal cells. Furthermore, a distinct motif derived from HIP1R has been demonstrated to stimulate the lysosomal degradation of *PD-L1* in cancer cells, presenting a novel opportunity for immunotherapy [154,155]. Nonetheless, the clinical application of CMA-based degraders is challenged by issues related to poor intracellular stability and ineffective delivery. From a mechanistic perspective, macroautophagy initiates with the formation of a phagophore, which is regulated by signaling complexes including *ULK1, Beclin1*, and *PI3K*. This is followed by the conjugation of *LC3-I*, resulting in the formation of *LC3-II*, a crucial step in the maturation of autophagosomes [156]. Ultimately, the fusion with lysosomes leads to the degradation of cargo within acidic compartments. This machinery is fundamental to all autophagy-targeting platforms, although each platform differs in its interaction with the autophagic system. Together, these innovative chemical biology tools represent a significant shift in targeted degradation, allowing induced proteostasis to extend beyond the limitations of the proteasome [157]. While every platform, *AUTACs*, *ATTECs*, *AUTOTACs*, and *CMA*-based chimeras, provides unique benefits, challenges concerning efficiency, selectivity, and mechanistic insights persist. Ongoing improvements in autophagy-inducing tags, receptor ligands, and linker chemistry, alongside advancements from structural biology and chemical proteomics, will be vital for progressing these approaches toward clinical use in cancer and other protein misfolding disorders Figure 15 [158].

Despite their conceptual promise, the autophagy-engaging platforms face substantial barriers to becoming drugs. AUTACs suffer from low potency, often requiring high micromolar concentrations that are difficult to achieve therapeutically without causing toxicity, and their mechanism remains unexplained [159]. ATTECs, while potent, are exceedingly difficult to discover due to the need to find small molecules that bind both *LC3* and an unrelated target with high affinity. AUTOTAC’s reliance on *P62* oligomerization risks disrupting a key node in cellular stress pathways, potentially leading to unpredictable side effects. Finally, all CMA-based strategies are currently hampered by the poor drug-like properties of peptides, including low metabolic stability and cell permeability, necessitating breakthrough advances and delivery technology [160].

## 6. Targeting the “Undruggable”: Structural Insights and Resistance Mechanisms

The transformative potential of PROTAC technology extends beyond improving existing drug targets; it offers a groundbreaking strategy to pursue proteins that are usually classified as “undruggable.” This category encompasses proteins that lack well-defined deep pockets or enzymatic activity crucial for high-affinity binding by traditional small-molecule inhibitors, including transcription factors, scaffold proteins, and GTPases. By functioning through an event-driven mechanism that requires only transient binding to induce degradation, PROTACs can effectively target these proteins based on surface epitope or protein–protein interactions, thereby vastly expanding the druggable proteome [6].

Key prospective target classes include oncogenic transcription factors such as *STAT3*, c-Myc, and mutant *p53*, which are pivotal drivers of tumorigenesis but also elude successful targeting due to their nuclear localization and largely flat interaction surfaces (Table 10). Similarly, *KRAS* mutants, e.g., G12D, G12C, among the most common oncogenic drivers in cancers like pancreatic, colorectal, and non-small cell lung carcinoma, present a smooth surface with picomolar affinity for GTP, making competitive inhibition extraordinarily difficult [161]. While allele-specific inhibitors for *KRAS12C* represent a major advance, resistance often emerges. PROTACs targeting *KRAS* could potentially degrade the protein completely, overcoming such resistance mechanisms. Furthermore, non-enzymatic scaffold proteins, which play a critical role in organizing signaling complexes, represent another compelling class of targets that are largely inaccessible to traditional occupancy-driven inhibitors [162].

Despite the promise, significant challenges remain. The primary barrier is the identification of a ligand, even a weak or non-functional one, that binds to a specific site on the target protein, which can then be incorporated into a PROTAC. For many undruggable targets, such ligands are still undiscovered [163]. Additionally, achieving sufficient degradation potency and selectively ensuring favorable pharmacokinetics for often complex molecules and avoiding on-target toxicities due to essential functions of some proteins in healthy tissues are critical barriers that must be overcome. Successfully degrading these intractable targets will require continued innovation in ligand discovery, linker optimization, and E3 ligase recruitment [164].

**Table 10:** Prospective target classes for PROTAC-based cancer therapy.

Target Class	Example Proteins	Rationale	Key Challenges	Ref.
Transcription Factors	STAT3, c-Myc, mutant p53	Master regulators of oncogenic gene expression; frequently mutated or overexpressed in cancers.	Flat protein–protein interaction surfaces; nuclear localization; lack of enzymatic activity.	[165,166]
GTPases	KRAS (G12D, G12C)	Among the most common oncogenic drivers; high unmet need in pancreatic, lung, and colorectal cancers.	Smooth protein surface with picomolar affinity for GTP; difficult to find competitive inhibitors.	
Scaffold Proteins	KSR1, ARID1A	KSR1: Scaffolds the Ras/Raf/MEK/ERK pathway. ARID1A: Critical subunit of the SWI/SNF complex; frequently mutated.	Often lack intrinsic catalytic activity, making conventional inhibitor screening impossible; bind partners via large, flat protein-protein interfaces that are difficult to disrupt with small molecules.	
Regulatory Subunits	BCAP, 14-3-3σ	BCAP: Regulates PI3K signaling. 14-3-3σ: Controls localization/activity of numerous client proteins (e.g., p53, FOXO).	Exist within large multi-protein complexes; degradation may require disassembly of the entire complex or may inadvertently stabilize it; functional redundancy among family members (e.g., other 14-3-3 isoforms) may limit efficacy.	

### 6.1. KRAS: From Inhibiting to Eliminating the Oncogene

The KRAS GTPase is mutated in approximately 25% of all cancers, with G12C, G12D, and G12V being the most common oncogenic drivers. Its smooth surface and picomolar affinity for GTP made it impervious to direct inhibition for decades. The breakthrough of allele-specific inhibitors (e.g., sotorasib for *KRAS(G12C*) that bind a newly discovered switch-II pocket (S-IP) adjacent to the mutant cysteine was a milestone. However, resistance rapidly emerges via secondary mutations (e.g., Y96D, R68S, H95D/Q/R) that impair drug binding or favor the GTP active state [167].

PROTAC targeting KRAS offers a compelling strategy to overcome this resistance by degrading the entire protein, thus overcoming any resistance mechanism reliant on its continued presence. The design of KRAS PROTACs hinges on using these same allele-specific inhibitors as POI ligands. for example, LC-2 is a KRAS(G12C)-targeting PROTAC based on the MRTX849 (adagrasib) scaffold linked to a VHL ligand. Structural studies and molecular dynamics simulations show that LC-2 forms a productive ternary complex with KRAS(G12C) and VHL, positioning the lysine residues on KRAS (e.g., *K104*, *K117*) within an optimal range for ubiquitination [168].

This degradation is effective even in cells with acquired resistance to MRTX849, as the mutations that prevent inhibitor binding do not necessarily prevent the initial transient engagement required for proteasome-induced ubiquitination. Recent efforts have also yielded PROTACs like HP5 based on a *pan-KRAS* inhibitor, showing efficacy against non-G12C mutants like G12D by degrading both wild-type and mutant *KRAS*, though with on-target toxicity challenges that necessitate refined targeting strategies [169].

### 6.2. Transcription Factors: c-Myc, STAT3, and Mutant p53

Transcription factors like *c-Myc, STAT3, and Mutant p53* are master regulators of oncogenic programs but lack traditional enzymatic pockets for high-affinity inhibitor binding [170].

c-Myc functions by heterodimerizing with Max. PROTACs have been designed to disrupt this protein–protein interaction (PPI). For instance, a PROTAC utilizing a Myc-Max PPI inhibitor (e.g., 10074-G5) linked to a CRBN and ligand demonstrated successful degradation of c-Myc.SAR studies revealed that linker length and composition were critical for degradation efficiency, with rigid, medium-length linkers optimizing the ternary complex formation between c-Myc, the PROTAC, and CRBN. Computational docking models suggest the PROTAC binds at the Myc-Max interface, and degradation leads to potent anti-tumor effects in vitro and in vivo [171].

STAT3 is activated by dimerization via its Src homology 2 (SH2) domain, while small molecule SH2 domain binders often have low affinity, they can be effective as PROTAC warheads. PROTACs like SD-36 (CRBN-based) achieve nanomolar-level degradation of STAT3. Crystallographic data of STAT3 inhibitor complexes inform the rational design of these PROTACs, ensuring the linker attachment point does not sterically hinder E3 ligase recruitment. SD-36 has shown efficacy in STAT3-dependent hematologic and solid tumor models, overcoming limitations of mere SH2 domain occupancy [172].

Mutant p53 often loses its tumor suppressive function and acquires oncogenic gain-of-function (GOF)properties. Stabilizing the misfolded mutant protein with chaperones like HSP70 is a key challenge. PROTAC such as MC886 (CRBN-based) successfully degrades common p53 mutants (R175H, R273H). The SAR demonstrates that PROTAC’s efficacy is dependent on the specific p53 binding moiety, with certain scaffolds preferentially engaging and degrading mutant over wild-type p53. This degradation depletes the GOF oncoprotein and can resensitize tumors to chemotherapy [173].

### 6.3. Overcoming Resistance: The Degrader Advantage

A key advantage of PROTAC is its potential to overcome resistance to traditional therapies.

Target Overexpression/Mutation, as seen with KRAS, resistance to inhibitors often involves upregulation of the target protein or point mutations at the binding site. Since PROTACs catalytically destroy the target, they can mitigate the effects of overexpression. Furthermore, mutations that reduce binding affinity for an inhibitor may not abolish it entirely, allowing the sub-stoichiometric PROTAC to still recruit the protein for degradation [38].

Bypassing Allosteric Resistance, in kinases like BTK, the C481S mutation abolishes binding for covalent inhibitors. PROTAC-like NX2127 can bind to alternative, non-catalytic sites (allosteric pockets) on BTK to mediate its degradation, effectively overcoming C481S-mediated resistance [174].

Addressing Scaffold Functions for proteins like IRK4, whose scaffolding role in Maddosome formation is critical for signaling, inhibition of its kinase activity is insufficient. PROTAC (e.g., KT-474) degrades the entire protein, ablating both enzymatic and scaffolding functions, which is a more comprehensive therapeutic strategy.

### 6.4. Albumin-Based Delivery Strategies

Expanding the scope of targeted protein degradation beyond conventional PROTAC design has increasingly focused on delivery optimization and non-oncological applications. Albumin-based delivery strategies have emerged as a promising approach to address key pharmacokinetic limitations of degraders, leveraging albumin’s long circulation, half-life, and tumor accumulation via enhanced permeability and retention effect. Both covalent and non-covalent albumin binding moieties have been explored to improve systemic exposure, reduce renal clearance, and enable controlled tissue distribution up to greater molecules. In parallel, targeted protein degradation is beginning to be investigated in infectious diseases, including exploratory efforts to degrade viral proteases and other essential viral proteins. Although still at an early stage, these approaches highlight the conceptual versatility of event-driven pharmacology and suggest that degrader-based strategies may ultimately extend beyond oncology into broader therapeutic areas where traditional inhibition has proven insufficient Table 11.

**Table 11:** Molecular strategies for targeting undruggable proteins with PROTAC.

Target Class	Example Protein	Structural Challenge	PROTAC Strategy and POI Ligand	Key SAR/Linker Insight	Resistance Bypass Mechanism	Refs.
GTPase	KRAS^G12C^	Smooth surface, high GTP affinity	S-IIP inhibitors (e.g., MRTX849-based)	Linker length critical for VHL/KRAS complex geometry.	Degrades protein, circumventing secondary point mutations (Y96D, R68S).	[175]
Transcription Factor	c-Myc	Lack of deep pockets, PPI-dependent	Myc-Max disruptors (e.g., 10074-G5 based)	Rigid, medium-length linkers optimize CRBN engagement.	Depletes master regulator, overcoming transcriptional addiction.	[176]
Signal Transducer	STAT3	The SH2 domain has a shallow surface	SH2 domain binders	Crystallographic data guide linker attachment point.	Degradation is more effective than SH2 occupancy for pathway blockade.	[177]
Mutant Tumor Suppressor	p53(R175H)	Misfolded, stabilized by chaperones	Small-molecule stabilizers/binders	Specific scaffolds favor mutant over wild-type p53 degradation.	Eliminates oncogenic GOF protein and chemoresistance.	[178,179]
Kinase/Scaffold	IRAK4	Kinase-independent scaffolding role	IRAK4 kinase inhibitors	N/A	Ablates both enzymatic and scaffolding functions.	[180]

## 7. Integration of Artificial Intelligence and Multi-Omics in PROTAC Development

The fusion of computational and molecular biology techniques is accelerating PROTAC innovation and development. The integration of artificial intelligence (AI) chemoinformatics and multi-omics is revolutionizing the field by optimizing degrader design, enabling site-specific target selection, improving patient classification, and ultimately advancing precision oncology [181].

### 7.1. Computational Design and Chemoinformatics

Researchers face significant challenges due to the structural complexity of PROTACS, which involves optimizing linker composition, cell permeability, and druglike properties. AI-powered chemoinformatic tools are increasingly deployed to solve these problems. These techniques aid in structure-activity relationship (SAR) modeling, molecular docking, and simulating ternary complex formation to inform the selection of target and E3 ligase ligands and linker design [182]. Machine learning models trained on large, curated datasets of PROTAC chemical structures and their associated degradation kinetics (DC_50_, Dmax9 can predict the degradation efficiency of novel compounds in silico, significantly accelerating the early-stage design cycle and synthesis process [183].

The emergence of specialized databases like PROTAC-DB has been instrumental in providing a foundational repository of experimentally validated degraders for model training. However, the predictive power of these models is constrained by the quality and volume of available data. Key pitfalls include overfitting to small or homogeneous datasets (e.g., models trained solely on CRBN-based degraders) and poor transferability to novel E3 ligase or target classes due to fundamentally different ternary complex dynamics. model validation against rigorous, standardized experimental assays is essential to benchmark accuracy and ensure generalizability [184].

### 7.2. Selection of Target and Ligase Using Omics Data

Mapping the “degradable proteome” relies heavily on multi-effect approaches, including proteomics, transcriptomics, and ubiquitinomics. Integrating gene and protein data from large patient cohorts (e.g., TGA) facilitates the identification of selective oncogenic dependencies that can be matched with available E3 ligases, enabling the design of tumor-selective PROTACs [185]. Mass spectrometry-based proteomics, such as activity-based protein profiling (ABPP) and thermal proteome profiling (TPP), enable proteome-wide monitoring of degrader selectivity and on and off-target effects [186].

Furthermore, spatial and single-cell transcriptomics reveal intratumoral heterogeneity in E3 ligase expression, guiding the design of PROTACs that are selectively active in specific cancer mutations and tumor microenvironments [187,188].

**Figure 16:**
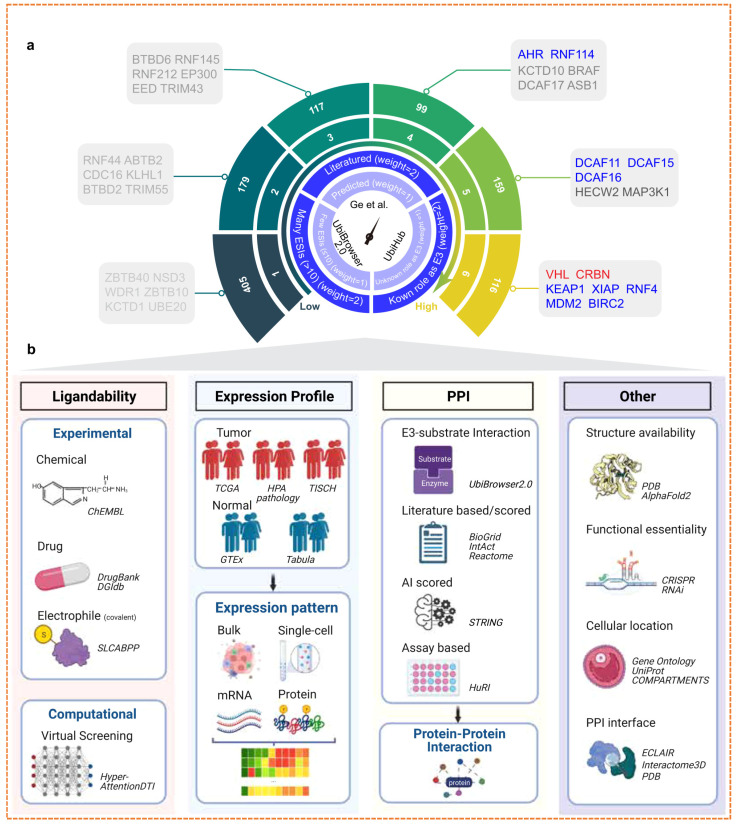
Schematic workflow of collecting and annotating E3 ligases. (**a**) Illustration of collecting and scoring E3 ligases in the inner circle, while the distribution of scores of E3 ligases and co-opted E3 ligases in the outer circle. E3 ligases in red were proceeded to PROTAC clinical trials. E3 ligases in blue were explored in the PROTAC experiment. (**b**) We comprehensively characterized E3 ligases chemical ligandability, expression patterns, protein–protein interactions (PPI), structure availability, functional essentiality, cellular location, and PPI interface. Reproduced with the permission of [29]. Copyright 2023 Nature.

Computational platforms like E3Atlas systematically prioritize E3 ligases for drug discovery by aggregating multi-omics criteria, including tissue-specific expression, essentiality scores, protein–protein interaction networks, and predicted ligandability, thus providing a data-driven strategy to expand the limited E3 ligase toolbox, as shown in Figure 16.

### 7.3. AI and Clinical Translation and Trial Design

AI is now transforming the clinical pathway of PROTAC technology. Resources like AlphaFold, PROTAC-DB, and E3-Atlas aid in the systematic, large-scale development of degraders by predicting ternary complex stability and prioritizing novel targets [11]. In clinical trials, AI-based tools are used to classify patients based on molecular profiles (e.g., high E3 ligase expression, low target redundancy), to estimate degradation efficiency, and correlate epigenetic/genetic alterations with treatment response. Furthermore, AI methods analyze complex data sets from single-cell RNA seq, circulating tumor DNA (ctDNA) [189], and imaging biomarkers to provide real-time feedback on degrader efficacy and the emergence of drug resistance. This capability is crucial for designing adaptive clinical trials and paving the way for truly personalized degrader therapies [190,191].

Despite this promise, clinical implementation constraints remain significant. The “black box” nature of the complex AI models can hinder regulatory acceptance. Furthermore, the ultimate clinical success of PROTACs will still be governed by traditional pharmacological challenges such as achieving adequate oral bioavailability and managing on-target toxicities that exist beyond the current predictive capabilities of AI. Overcoming these hurdles will require close collaboration between computational and experimental scientists to generate robust standardized data sets for model training and to develop interpretable AI that can earn the trust of clinicians and regulators [192].

## 8. Future Directions and Conclusions

The clinical translation of PROTACs marks a significant transition in precision oncology, transforming targeted protein degradation from a largely conceptual strategy into a clinically validated therapeutic modality for treatment-resistant cancers. Early clinical candidates such as the androgen receptor degrader ARV-110, the estrogen receptor degrader ARV-471, the BCL-xL degrader DT2216, and the dual BTK/IKZF1/3 degrader NX-2127 have collectively provided important lessons that extend beyond proof-of-concept efficacy. These studies have demonstrated that sustained target suppression can be achieved through catalytic degradation in humans, that complete target elimination is not always required for clinical benefit, and that acceptable tolerability profiles are possible despite the large molecular size of degrader molecules. Together, these insights distinguish PROTACs fundamentally from occupancy-driven inhibitors and highlight their potential to address acquired resistance, limited safety margins, and undruggable targets. Despite this progress, the path to widespread clinical adoption remains challenging. The intrinsic physicochemical properties of PROTACs, including high molecular weight, conformational flexibility, and lipophilicity, continue to complicate synthesis, oral bioavailability, and tissue penetration. In parallel, emerging resistance mechanisms such as alterations in E3 ligase expression or target accessibility raise important questions about the long-term durability of degrader therapies. Among these challenges, the limited availability of tissue-selective E3 ligases remains a central bottleneck, as most clinically advanced PROTACs still rely on CRBN or VHL, increasing the risk of on-target but off-tissue toxicity. Future advances and targeted protein degradation will therefore depend on coordinated innovation across chemistry, biology, and clinical strategy. Next-generation degraders are being designed with improved drug-like properties, enhanced selectivity, and greater control over tissue specificity. Expanding the repertoire of ligands for previously unexplored E3 ligases, particularly those with restricted expression patterns, represents a critical opportunity to improve therapeutic precision and mitigate toxicity. In parallel, Chemo proteomics and AI-assisted molecular design are increasingly used to identify degrader-amenable targets, optimize ternary complex formation, and predict degradation efficiency in a context-dependent manner. At the clinical level, combination strategies are emerging as a rational approach to maximize the therapeutic impact of PROTACs. By integrating degraders with immune checkpoint inhibitors, kinase inhibitors, or DNA damage targeting agents, it may be possible to exploit network-level vulnerabilities, delay resistance, and reshape the tumor microenvironment. Equally important are advances in delivery technologies, including tumor-activated pro-drug strategies, PROTAC-antibody conjugates, and stimulus-responsive nano-PROTACs, which aim to enhance tissue selectivity while minimizing systemic exposure. Human development approaches incorporating biomarker-guided patient selection, multi-omics profiling, and predictive modeling are expected to play an increasingly central role in guiding clinical trial design and enabling personalized degrader therapies. In conclusion, PROTAC Technology has fundamentally expanded the druggable proteome by enabling the selective elimination of disease-causing proteins rather than their transient inhibition. While challenges related to chemistry, delivery, and selectivity remain, the rapid pace of innovation in ligand discovery, E3 ligase biology, and translational strategies suggest a realistic path forward. Continued progress will depend not only on technological refinement but also on a deeper understanding of degradation biology and human systems. If these challenges are met, targeted protein degradation is poised to deliver durable therapeutic solutions across oncology and potentially, immune and neurodegenerative diseases that have long resisted conventional pharmacological approaches.

## Figures and Tables

**Table 4:** Selected PROTACs in Clinical Trials for Cancer.

PROTAC Candidate	Target Protein	Indication	Developer	Mechanism of Action	Trial Phase	Trial Identifier	Clinical Status	Ref.
**ARV-110**	Androgen Receptor	Metastatic Castration-Resistant Prostate Cancer (mCRPC)	Arvinas	Induces AR degradation via VHL recruitment	Phase I/II	NCT03888612	Demonstrated PSA reduction and partial responses	[75]
**ARV-471**	Estrogen Receptor α	ER+/HER2− Metastatic Breast Cancer	Arvinas and Pfizer	ERα degradation via VHL E3 ligase	Phase II/III	NCT04072952	Safe and well-tolerated, showing antitumor activity	[76]
**DT2216**	BCL-xL	Hematologic Malignancies (e.g., T-cell lymphoma)	Dialectic Therapeutics	BCL-xL degradation using VHL-based PROTAC	Phase I	NCT04886622	Overcomes thrombocytopenia seen in BCL-xL inhibitors	[77]
**NX-2127**	BTK + IKZF1/3	Chronic Lymphocytic Leukemia (CLL), B-cell malignancies	Nurix Therapeutics	Dual degradation via CRBN; BTK + transcription factors	Phase I	NCT04830137	Oral PROTAC, early efficacy in BTK-inhibitor-resistant CLL	[78]
**ACBI1**	BRD9	Solid Tumors and Synovial Sarcoma	C4 Therapeutics	BRD9 degradation via E3 ligase binding	Preclinical–IND	N/A	High selectivity: preclinical proof-of-concept established	[79]

Abbreviations: AR—Androgen Receptor; ER—Estrogen Receptor; BTK—Bruton’s Tyrosine Kinase; CLL—Chronic Lymphocytic Leukemia; CRBN—Cereblon; VHL—Von Hippel-Lindau E3 ligase; IND—Investigational New Drug application.

**Table 5:** Comparative Physicochemical and Pharmacokinetic Properties of Selected Clinical Stage PROTACs.

PROTAC	Target	Molecular Weight (Da)	Calculated clogP	Clinical Phase	Key PK Finding (Dose)	Reference (Trial Source)
**ARV-110**	AR	~1000	~6.5	Phase II	Mean t_1_/_2_ ≈ 3.6 h (420 mg QD)	[NCT03888612]
**ARV-471**	ERα	~1000	~5.8	Phase III	Mean t_1_/_2_ ≈ 12–18 h; high oral bioavailability	[NCT04072952]
**KT-474**	IRAK4	~900	~4.2	Phase I	Dose-dependent exposure; sustained target engagement	[NCT04772885]
**DT2216**	BCL-xL	~1100	~7.1	Phase I	No significant thrombocytopenia at efficacious doses	[NCT04886622]
**NX-2127**	BTK	~1000	~5.5	Phase I	Rapid, sustained BTK degradation in patient PBMCs	[NCT04830137]

Abbreviations: t_1_/_2_, half-life; QD, once daily; PBMCs, peripheral blood mononuclear cells.

**Table 6:** Representative process risks and mitigation strategies for PROTAC manufacture.

Failure Mode	Root Cause	Mitigation/Control	Critical Metric	Ref.
Linker hydrolysis/instability	Labile ester, carbonate, PEG oxidation	Favor amide/urea linkages; water/peroxide control; stress-test linker fragments	Stability (HPLC/LC–MS), % degradation	[105]
Low-yield amide coupling	Steric hindrance, O→N acyl shift	Screen coupling reagents, DoE optimization, flow amidation	Step yield, impurity profile	[106]
Isomer/atropisomer mixtures	Multiple stereocenters, aryl–aryl linkers	Chiral pool synthesis; asymmetric catalysis; atropisomer locking	Enantiomeric excess, diastereomer ratio	[107]
Impurity carryover	Over-acylation, oligomerization, Cu residues	In-process purge maps; flow CuAAC; orthogonal LC–MS checks	Impurity levels vs. ICH Q3A/B	[108]
Scale-up failure (oiling-out, filtration issues)	Gummy, hygroscopic solids; high polarity	Anti-solvent crystallization, spray/freeze drying, salt/co-crystal forms	Solid form stability, % recovery	[109]
GMP compliance delays	Non-green solvents, complex analytics	Early solvent swaps; validated orthogonal methods (qNMR + LC–MS + chiral LC)	Release timeliness, method transfer	[110]

**Table 7:** Key Challenges and Potential Solutions in PROTAC Development.

Challenge	Description	Potential Solutions	Ref.
**Poor Pharmacokinetics and Bioavailability**	High molecular weight and polarity limit oral absorption and systemic exposure.	Prodrug strategies, nanoparticle formulations, subcutaneous/intravenous delivery, and linker optimization.	
**Limited Cell and Tissue Penetration**	Large size restricts membrane permeability; the blood–brain barrier (BBB) limits brain targeting.	Use of cell-penetrating peptides, BBB-permeable designs, and carrier-based delivery systems.	[112]
**E3 Ligase Expression Constraints**	CRBN and VHL have restricted expression; mutations may cause resistance.	Discovery of novel E3 ligases (e.g., DCAF16, RNF4), dual ligase recruiting PROTACs, and ligand ability mapping.	[113]
**Off-target Degradation and Toxicity**	Unintended degradation due to promiscuous E3 ligase interactions.	Use of degraded selectivity profiling, high-resolution structural modeling, and tissue-specific ligases.	[114]
**Acquired Resistance Mechanisms**	Target mutations or loss of E3 ligase activity impair PROTAC activity.	Development of backup ligands, dual-degrading strategies, and target-switching PROTACs.	[115]
**Chemical Complexity and Scalability Issues**	Bifunctional structure complicates synthesis, stability, and mass production.	Modular synthetic platforms, simplified linkers, automated synthesis pipelines.	[116]
**Immunogenicity and Systemic Effects**	Degradation of immune regulators may provoke unintended immune responses.	Early-stage immunoprofiling, target selection filters, and combination with immune checkpoint inhibitors.	[114]

**Table 8:** Emerging E3 ligases for PROTAC development: A comparative analysis of ligandability and translational potential.

E3 Ligase	Known Ligands and Chemotypes	Tissue/Cancer Specificity	Validated Targets	Key Advantages	Major Challenges for Translation	Ref.
**RNF114**	Zinc-ejectors (e.g., disulfiram), covalent fragments	Overexpressed in squamous cell carcinomas	BRD4, ERK1/2	Tissue-specific overexpression offers a therapeutic window; cysteine-rich for covalent discovery.	Risk of off-target effects via zinc ejection; potential immunogenicity.	[110,122].
**KEAP1**	Electrophilic fragments, NRF2 peptide mimetics	High in lung, breast cancer	NRF2, STING	Well-characterated pocket; potential for TME-activated PROTACs.	High endogenous levels may cause competition; risk of NRF2 pathway activation.	[123,124]
**DCAF16**	Cysteine-directed covalent fragments (chloroacetamides)	Broad	Nuclear proteins (BRD4)	Small size; validated by chemoproteomics.	Limited knowledge of native substrates; off-target cysteine reactivity.	
**SOCS2/5**	Peptide mimetics (under development)	Immune cells, hematological malignancies	JAK2, STATs	Key for immuno-oncology targets; inherent specificity.	Potent, drug-like small-molecule ligands are not yet fully validated.	[114,115].
**FBXO22**	Small-molecule ligands (recently discovered)	Overexpressed in glioblastoma, melanoma		Potential for targeting CNS and skin cancers.	Novel target; degradation efficacy not yet broadly proven.	

**Table 9:** Comparison of novel degradation modalities.

Technology	Mechanism of Action	Key Advantage	Representative Target	In Vivo Efficacy Evidence	Major Limitations and Translational Hurdles	Ref.
**Molecular Glues**	Monovalent small molecules that stabilize the interaction between an E3 ligase and a neosubstrate.	Excellent drug-like properties (low MW, good bioavailability); no linker required.	IKZF1/3, GSPT1	Clinical validation with drugs like lenalidomide and CC-885 in hematologic cancers.	Discovery is largely serendipitous; rational design remains challenging. Limited to targets amenable to induced proximity.	[131]
**Trivalent PROTACs (e.g., TriTACs)**	Incorporates a third binding moiety to stabilize the ternary complex (POI-PROTAC-E3).	Enhanced cooperativity and selectivity for complex targets.	BRD4	SIM1 degrades BRD4 at picomolar concentrations in cells	Extremely high molecular weight (>1200 Da) compromises oral bioavailability. Synthetic complexity hinders large-scale manufacturing.	[132]
**LYTACs**	Recruits cell-surface lysosomal shuttling receptors (e.g., CI-M6PR, ASGPR) to degrade extracellular and membrane proteins.	First modality to target the extracellular proteome.	EGFR, PD-L1	Cetuximab-GalNAc LYTAC degraded >70% of EGFR in xenograft models	Immunogenicity risk from antibody/foreign glycan components. Limited tissue targeting (e.g., GalNAc is liver-specific). Poor tissue penetration of large conjugates.	
**AUTACs**	S-guanylation tag (FBnG) mimics a “eat-me” signal for macroautophagy.	Can degrade organelles (e.g., mitochondria) and protein aggregates.	Mitochondria, FKBP12	Induced mitophagy in Down syndrome cell models and in vivo	Low potency (requires high µM concentrations). Poorly understood mechanism. Lacks catalytic efficiency.	[133]
**ATTECs**	“Molecular glue” that directly binds both the target and LC3 on autophagosomes.	High specificity; can distinguish mutant from wild-type protein.	mHTT, lipid droplets	Compound 105 reduced mHTT aggregates in a Huntington’s disease mouse model	Screening challenge: discovering dual binders is difficult. Limited and narrow target scope.	[134]
**CMA Chimeras**	Peptide-based chimera with a CMA targeting motif (e.g., KFERQ).	Potential for high selectivity via peptide-POI binding.	PD-L1	Degraded PD-L1 in cancer cells via a HIP1R-derived motif	Peptide-based: Poor stability, low permeability, rapid clearance. Delivery is a major unsolved problem.	

## Data Availability

This manuscript does not report data generation or analysis.

## Abbreviations

The following abbreviations are used in this manuscript:

PROTAC	Proteolysis-Targeting Chimera
UPS	Ubiquitin–Proteasome System
E3	E3 Ubiquitin Ligase
CRBN	Cereblon
VHL	Von Hippel–Lindau Protein
MDM2	Mouse Double Minute 2 Homolog
LYTAC	Lysosome-Targeting Chimera
RNA	Ribonucleic Acid
DNA	Deoxyribonucleic Acid
TCGA	The Cancer Genome Atlas
PPIs	Protein–Protein Interactions
AI	Artificial Intelligence
PK/PD	Pharmacokinetics/Pharmacodynamics
CNS	Central Nervous System
TPD	Targeted Protein Degradation
FDA	Food and Drug Administration
IND	Investigational New Drug
SAR	Structure–Activity Relationship
SPR	Surface Plasmon Resonance
ADME	Absorption, Distribution, Metabolism, and Excretion
KEAP1	Kelch-like ECH-associated Protein 1
DCAF16	DDB1- and CUL4-Associated Factor 16
UHRF1	Ubiquitin-like with PHD and RING Finger Domains 1
SCF	Skp1–Cullin–F-box Complex
DUB	Deubiquitinating Enzyme
BRD4	Bromodomain-Containing Protein 4
BCL-xL	B-Cell Lymphoma-Extra Large
CDK	Cyclin-Dependent Kinase
KRAS	Kirsten Rat Sarcoma Viral Oncogene
EGFR	Epidermal Growth Factor Receptor
